# Ethambutol induces optic neuropathy through SDHB-mediated ferroptosis in retinal ganglion cells via Smad4 pathway

**DOI:** 10.1007/s13577-025-01342-4

**Published:** 2026-01-21

**Authors:** Qiushi Li, Wei Ge, Yifan Zhang, Qibin Xu, Junli Xu, Yu Zhang, Xingneng Guo, Wenyan Sheng, Liwei Zhu

**Affiliations:** Department of Ophthalmology, Hangzhou Red Cross Hospital, No. 208, Huancheng East Road, Gongshu District, Hangzhou, 310000 Zhejiang China

**Keywords:** Ethambutol, Optic neuropathy, Ferroptosis, SDHB, Retinal ganglion cells

## Abstract

Ethambutol (EMB)-induced optic neuropathy (EON) is a clinical concern. Ferroptosis, involving iron and toxic reactive oxygen species (ROS), causes unique cell death, but its mechanism in EON is unclear. This study aims to explore the EON mechanisms. Wistar rats were used to establish an EON model by administering EMB at 50 mg/kg daily for 8 weeks. Retinal ganglion cells (RGC-5 cells) were used for in vitro experiments. Histological staining, MTT assays, flow cytometry, western blot analysis, dual-luciferase reporter assay, chromatin immunoprecipitation, and high-throughput sequencing were conducted to investigate cell death modes and molecular changes. EMB treatment leads to significant cell loss and structural damage in RGCs of EON model, predominantly through ferroptosis. We confirm increased ROS levels, downregulation of SLC7A11 and GPX4, and decreased glutathione (GSH) levels, upregulation of malondialdehyde (MDA) and 4-hydroxynonenal (4-HNE) levels in EMB-treated RGC-5 cells. Furthermore, sequencing data reveal that in RGC-5 cells treated with EMB, the differentially expressed genes (DEGs) primarily exhibited alterations in biological functions associated with metabolism, stress response, and apoptotic regulation. Specifically, EMB inhibits the expression of succinate dehydrogenase enzyme B (SDHB), thereby disrupting antioxidant defenses and facilitating ferroptosis. Moreover, Smad4 has been pinpointed as a pivotal transcription factor in regulating SDHB expression. Notably, its interaction with the promoter region of SDHB is inhibited by EMB. This study provides compelling evidence for the involvement of ferroptosis in EON and highlights SDHB and Smad4 as potential therapeutic targets for mitigating this adverse effect.

**Supplementary Information:**

The online version contains supplementary material available at 10.1007/s13577-025-01342-4.

## Introduction

Ethambutol (EMB), a frontline antibacterial drug, is crucial for treating *Mycobacterium* tuberculosis infections by inhibiting the arabinosyltransferase enzyme in mycobacteria [1, 2]. However, a significant drawback of EMB therapy is the potential for optic neurotoxicity, causing irreversible vision loss in some patients, known as ethambutol-induced optic neuropathy (EON), featuring blurred vision, color vision disturbances, and central or cecocentral scotomas [3]. Typically, ocular symptoms emerge after 7–8 months of EMB treatment, and the development of EON is both time- and dose-dependent. Risk factors for EON include advanced age, low body weight, and renal insufficiency [4]. Although cessation of EMB may lead to reversal of visual impairment in some cases, severe or permanent vision loss can occur even with standard doses [5], hinting at potential additional predisposing factors.

EON is often diagnosed late due to challenges in early detection through subjective examinations, such as visual acuity, color vision testing, and visual field monitoring [6]. Previous studies have reported that 1–2% of patients administered EMB doses ranging from 15 to 25 mg/kg/day may develop EON [7, 8]. Thus, quantitative, objective biomarkers are urgently needed to facilitate early detection of EON. Recent research on the pathophysiology of EON remains largely clinical [9, 10], with Optical coherence tomography (OCT) measuring peripapillary retinal nerve fiber layer (p-RNFL) thickness showing promise for early EON diagnosis [5, 11]. However, a definitive standard is yet to be established. Our preliminary studies have shown that the ganglion cell-inner plexiform layer (GCIPL) thickness, measured by OCT, decreases before p-RNFL thinning in EON patients, suggesting direct damage to the retinal ganglion cell (RGC) layer by EMB [12].

Increasing evidence points to neurodegeneration of the innermost retinal neurons, specifically the RGCs and their axons (forming the optic nerve), as a hallmark of optic neuropathies. Oxidative stress, including oxidative damage and mitochondrial dysfunction, is likely a critical pathogenic mechanism [13–15]. Thus, the question arises: how does EMB exert its biological effects in EON? Understanding the underlying mechanisms of EMB-induced neurotoxicity is crucial for developing effective strategies to prevent or mitigate EON. Therefore, this study aims to elucidate the intricate interactions between EMB and retinal neurons, particularly focusing on oxidative stress pathways and potential neuroprotective interventions.

## Materials and methods

### Cell lines and cell culture

The RGC-5 (YDT-0728) cell lines used in this study were obtained from INDIT Bio-Technology Co., Ltd (Hangzhou, China) and cultured in DMEM/F-12 complete medium (IMMOCELL, Xiamen, China, IMC-305) with 10% fetal bovine serum (FBS) and 1% penicillin–streptomycin (PS) in a 37 °C, 5% CO_2_ incubator (Thermo, USA, NO. 311). When the cells reached 80–90% confluence, they were subcultured at a ratio of 1:2, and cells in the logarithmic growth phase were selected for experiments. Cell lines used in this study were authenticated by short tandem repeat (STR) profiling and confirmed to be free of mycoplasma contamination using a PCR-based detection method. The RGC-5 cell line used in this study has been reclassified as a rat retinal precursor cell line [16, 17], though it remains a widely utilized model in retinal disease research.

### Construction of EON rat model

This study’s animal experimentation procedures strictly adhered to the Guiding Principles for the Breeding and Use of Animals in China, ensuring ethical and humane animal treatment. Twenty 6-week-old male Wistar rats were purchased from Vital River Laboratory Animal Technology Co., Ltd. (Beijing, China) and acclimatized for 1 week in a controlled condition (pathogen-free, day/night: 12/12, 22 °C) to minimize stress before experiments. After acclimatization, rats were randomly divided into control and experimental groups (10 rats/group). The experimental group received 50 mg/kg EMB daily via intragastric administration for 8 weeks, and the control group received an equal volume of saline on the same schedule. This well-established EON model has been widely used in ophthalmic research [13, 18]. After treatment, rats were euthanized; one eye from each rat was fixed in a universal tissue fixative (Servicebio, Wuhan, China, G1101-3ML) to make slices for histological analyses, while the contralateral retina was snap-frozen and stored at − 80 °C for molecular biology experiments.

The animal experiments were conducted by the “Guiding Principles in the Care and Use of Animals” (China) and approved by Shengyuan Life Sciences and Technology (Anji) Co., Ltd (approval number: 2025003).

### Hematoxylin and eosin (H&E) staining

Selected slices were stained with a hematoxylin and eosin staining kit (Beyotime, Shanghai, China, C0105M) following standard protocols for histological evaluation. Briefly, slices were dewaxed in xylene (SINOPHARM, Beijing, China, 10023418) and rehydrated through a graded series of ethanol solutions. They were then stained with hematoxylin for 10 min to impart a blue color to nuclei, followed by eosin staining for 2 min to stain cytoplasm and extracellular matrix pink. After staining, the slides were dehydrated in ethanol, cleared in xylene, and sealed with a permanent mounting medium (SINOPHARM, 10004160). The stained slices were observed under a microscope (Nikon, Japan, E100) for morphological assessment and the images were collected and analyzed.

### Immunofluorescence (IF) staining

For IF analysis, dewaxed and rehydrated slides were washed 3 times with PBS (PH 7.4) for antigen retrieval at 5 min each time. The slices were placed in 3% hydrogen peroxide (ANNHET^®^, Shangdong, China, D.40036) in methanol for 10 min, and then incubated with 3% bovine serum albumin (BSA) for 30 min at room temperature. Primary antibodies specific to RNA-binding protein with multiple splicing (RBPMs, Proteintech, Wuhan, China, 15187-1-AP), an RGCs marker antibody [19], were diluted 1:200 and incubated overnight at 4 °C. After washing with PBS, slides were incubated with horseradish peroxidase (HRP)-conjugated secondary antibodies (CST, Danvers, MA, USA, 7074P2) for 50 min at room temperature, followed by incubating with DAPI staining solution (Servicebio, G1012) at room temperature and away from light for 10 min. After adding the antifade mounting medium (Servicebio, G1401) to the slide, the images were observed and collected using a fluorescence microscope (Olympus, Tokyo, JP, CKX53).

### Methylthiazolyldiphenyl-tetrazolium bromide (MTT) assay

MTT assays detected cell proliferation and cytotoxicity in treated RGC-5 cells. In brief, logarithmic growth-phase cells were suspended and seeded at 2 × 10^3^ cells/well in 96-well plates (100 μL). For proliferation, cells were incubated for 0, 24, 48, and 72 h post-transfection and EMB treatment. For cytotoxicity, cells were exposed to different concentrations of EMB (0, 1, 2, 4, 8 mM) for 12 and 24 h or 2 mM EMB combined with Chloroquine (CQ, 20 μM), Ferrostatin-1 (Fer-1, 2 μM), and VX-765 (40 μM). MTT reagent (1 mg/mL) (Sigma-Aldrich, St. Louis, MO, USA, M2128) was added at each timepoint (50 μL), incubated for 3 h at 37 °C, and then dissolved with dimethyl sulfoxide (DMSO) (150 μL/well). Absorbance at 570 nm was measured using a microplate reader (Thermo, 51119570) and then analyzed.

### Flow cytometry assay

Flow cytometry was implemented to measure lipid and total reactive oxygen species (ROS) levels in RGC-5 cells after pretreatment. According to the manufacturer’s protocol, a ROS Assay Kit (Beyotime, Shanghai, China, S0033S) with the fluorescent probe DCFH–DA was employed. Briefly, cells were incubated with DCFH–DA diluted 1:1000 in serum-free medium at 37 °C for 20 min, followed by immediate ROS detection using flow cytometry (Attune NxT, Invitrogen, Waltham, USA).

### Western blotting (WB) assay

WB assays were conducted to detect protein expression. In brief, total proteins from pretreated cells or retinal tissues were extracted and quantified using a BCA assay kit (Takara, Beijing, T9300A) at 570 nm absorbance. Next, 20 µg of protein was separated by 10% SDS–PAGE (Sigma-Aldrich, 74255) using electrophoresis (200 V constant pressure) for 1 h and transferred to a nitrocellulose membrane (Merck Millipore, Billerica, MA, USA, IPVH00010). The membrane was blocked with 5% BSA for 2 h, followed by 4 °C incubations with primary antibody overnight and 2-h secondary antibody incubations at 37 °C. Protein bands were visualized using an ECL kit (Biosharp, Beijing, China, BL520B). Data analysis results are presented as a bar graph, normalized to β-actin. Antibody details are provided in Table 1.

**Table 1:** Details of antibodies

Name	Brand	Number
SLC7A11	Abcam	15187-1-AP
GPX4	ABclonal	ab216876
β-Actin	ABclonal	AC026
SDHB	Proteintech	10620-1-AP
p-NFκB	CST	3033T
NFκB	CST	8242T
p-Smad2/3	CST	8828S
Smad2/3	CST	8685T
p-Smad4	Affinity	AF8316
Smad4	CST	38454S
p-STAT4	Affinity	AF3441
STAT4	Proteintech	67568-2-Ig
Anti-rabbit IgG, HRP-linked antibody	CST	7074P2
Anti-mouse IgG, HRP-linked antibody	CST	7076P2

### Real-time reverse transcription PCR (RT-qPCR) assay

The total RNA of the treated RGC-5 cells was extracted with 100 μL Trizol reagent (Ambion, USA 15596018). Subsequently, 100 ng of total RNA was used for the reverse transcription process, employing a cDNA synthesis kit (Thermo, K1622). The obtained cDNA was then used to perform qPCR using an ABI-7500 system (Thermo, 7300). The relative expression levels of the mRNA were determined using the 2^−ΔΔ*Ct*^ method. The specific primers used in this qPCR analysis are detailed in Table 2.

**Table 2:** Details of primer sequences

Name	Sequence
Cox6a2	F: CTGACCTTTGTGCTGGCTC R: GTGATACGGGATGAACTCTGG
Uqcrq	F: CTGACCTTTGTGCTGGCTCT R: GTGATACGGGATGAACTCTGG
Cox5a	F: CCGCTGTCTGTTCCATTC R: TGAGGTCCTGCTTTGTCC
SDHB	F: GAAGGCATCTGTGGCTCT R: TGGATTTGTATTGTGCGTAG
Ndufb9	F: TTCCAGAATGGTGCTTAG R: TCAGGTGATGTTTCCTCC
Ndufa7	F: AGTTGTGCCTCCCTCAATC R: GGTGTCACTGCCTTCTTCTC

### Detection of GSH, MAD, and 4-HNE levels

To investigate the levels of glutathione (GSH), malondialdehyde (MDA), and 4-hydroxynonenal (4-HNE), indicators of ferroptosis, in the treated RGC-5 cells, cells were first cultured under standard conditions until they reached the logarithmic growth phase. Cells were then harvested and processed to form a cell suspension. The expression levels of GSH, MAD, and 4-HNE in RGC-5 cells were, respectively, detected by the Mouse GSH ELISA kit (MEIMIAN, Jiangsu, China, MM-0661M2), Mouse MDA ELISA kit (MEIMIAN, MM-0897M2), and Mouse 4-HNE ELISA kit (MEIMIAN, MM-43796M1) according to the kit’s instructions.

### High-throughput sequencing and analysis

Total RNA was extracted and collected from the logarithmic growth of RGC-5 cells (control group) and RGC-5 cells were treated with 4 μM EMB for 24 h (experimental group). The study included 8 samples (4 control and 4 EMB-treated) with each yielding 41–51 million clean paired-end reads (6.1–7.6 Gb clean bases) and Q30 scores of 96.6–97.1%. Libraries were prepared using Oligo(dT) bead enrichment followed by fragmentation, double-stranded cDNA synthesis, end repair, A-tailing, adapter ligation and PCR amplification. Sequencing reads were aligned to the Ensembl reference genome using HISAT2 (v2.0.5). The samples were collected for high-throughput sequencing analysis that was conducted on LC–Bio Technologies (Hangzhou, China) to identify differentially expressed genes (DEGs). Differential expression analysis was performed using DESeq2 (v1.20.0) with Benjamini–Hochberg FDR correction, applying thresholds of *p* value ≤ 0.05 and |log₂FoldChange|≥ 0. The results were visualized in heatmaps and volcano plots, where the volcano plot combined fold change (log_2_FC) and statistical significance (− log10(*p* value) to identify genes with significant expression changes. Gene Ontology (GO) and Kyoto Encyclopedia of Genes and Genomes (KEGG) enrichment analyses were conducted on these DEGs to understand their biological roles and to identify enriched pathways. A Venn diagram was used to depict the overlapping genes in significant enrichment pathways. Using the intersection gene expression profile, we generated a heatmap with the “complexheatmap package” of R software. Furthermore, hierarchical clustering was applied to group genes based on their fragments per kilobase of transcript per million mapped reads (FPKM) values.

### Transient transfection

For transiently transfected cells, we downloaded succinate dehydrogenase enzyme B (SDHB, NM_023374) and Smad4 (NM_008540) transcripts from the NCBI database (https://www.ncbi.nlm.nih.gov/). Their overexpressed plasmids, sourced from Youbio (Hunan, China), were cloned onto pcDNA3.1 or pCDH vector to construct recombinant plasmids. For SDHB silencing in RGC-5 cells, Tsingke Biotechnology (Beijing, China) provided the plasmids of siRNAs targeting the SDHB (siR-SDHB) and the control siRNAs (siR-NC). When RGC-5 cells reached 80% confluence, they were treated overnight with the serum-free medium before transfection with the plasmids using Lipofectamine 2000 (Invitrogen, MA, USA, 11668-019) and Opti-MEM (Gibco, Grand Island, NY, USA, 31985-070) for 24 h. Finally, the transfection efficiency in RGC-5 cells was confirmed by WB assays. Sequence details of siRNA information are listed below:

siR-NC-sense: UUCUCCGAACGAGUCACGUTT

siR-NC-antisense: ACGUGACUCGUUCGGAGAATT

siR-SDHB-1-sense: CCCUGAUUUGAGUAACUUCUA

siR-SDHB-1-antisense: UAGAAGUUACUCAAAUCAGG

siR-SDHB-2-sense: GACUGGAGAUAAACCUCGAAU

siR-SDHB-2-antisense: AUUCGAGGUUUAUCUCCAGUC

siR-SDHB-3-sense: GAAAGCGAUUGCGGAAAUCAA

siR-SDHB-3-antisense: UUGAUUUCCGCAAUCGCUUUC

### Dual-luciferase reporter assay

To validate the regulatory relationship, a dual-luciferase reporter assay was conducted. SDHB wild-type (SDHB-Wt) and mutant (SDHB-Mut) plasmids, sourced from Hippo Bio (Hangzhou, China), were cloned into PGL3 vectors. These plasmids and controls were transfected into RGC-5 cells with or without 2 mM EMB treatment using Lipofectamine 2000 for 24 h. Luciferase activity was then measured using a Dual-Luciferase^®^ Reporter Assay kit (Promega, Madison, WI, USA, E1910) according to the manufacturer’s specifications. Specific sequence information is shown in the supplementary information.

### Chromatin immunoprecipitation (ChIP)

RGC-5 cells treated with 2 mM EMB and controls were cross-linked with methanol at 37 °C for 10 min to form protein–DNA complexes, following CHIP-Seq High Sensitivity Kit instructions (Abcam, Cambridge, UK, ab185908). Cells were then sonicated in an ultrasonic cell crusher (SCIENTZ, Ningbo, China, JY92-IIN) to obtain DNA fragments. Supernatants were diluted, with 40 μL of control added to a 1.5 mL tube as an Input group. Both control and experimental groups were incubated with Smad antibody at 4 °C overnight, followed by rProtein A/G MagPoly beads (Smart-Lifesciences, Changzhou, China, SM015005) for 1 h. Complexes were collected, DNA purified using a Clean-Up Kit (Omega, D6296-01), and SDHB promoter fragment enrichment in each group was validated by RT-qPCR (F primer: TCTGAGTTCAAGTCCAGTCTG; R primer: TGTCCTCAGATTTCGTCAGTT).

### Statistical analysis

All experiments were independently performed at least three times to guarantee repeatability. A statistical analysis of the representative data was conducted utilizing the GraphPad Prism 6.0 software. Specifically, statistical comparisons between two distinct groups were assessed using the *t* test method. A one-way ANOVA test was employed for evaluations involving multiple groups. The threshold for determining statistical significance was set at a *p* value of less than 0.05.

## Results

### EMB induces optic neuropathy through ferroptosis-mediated mechanisms

**Fig. 1:**
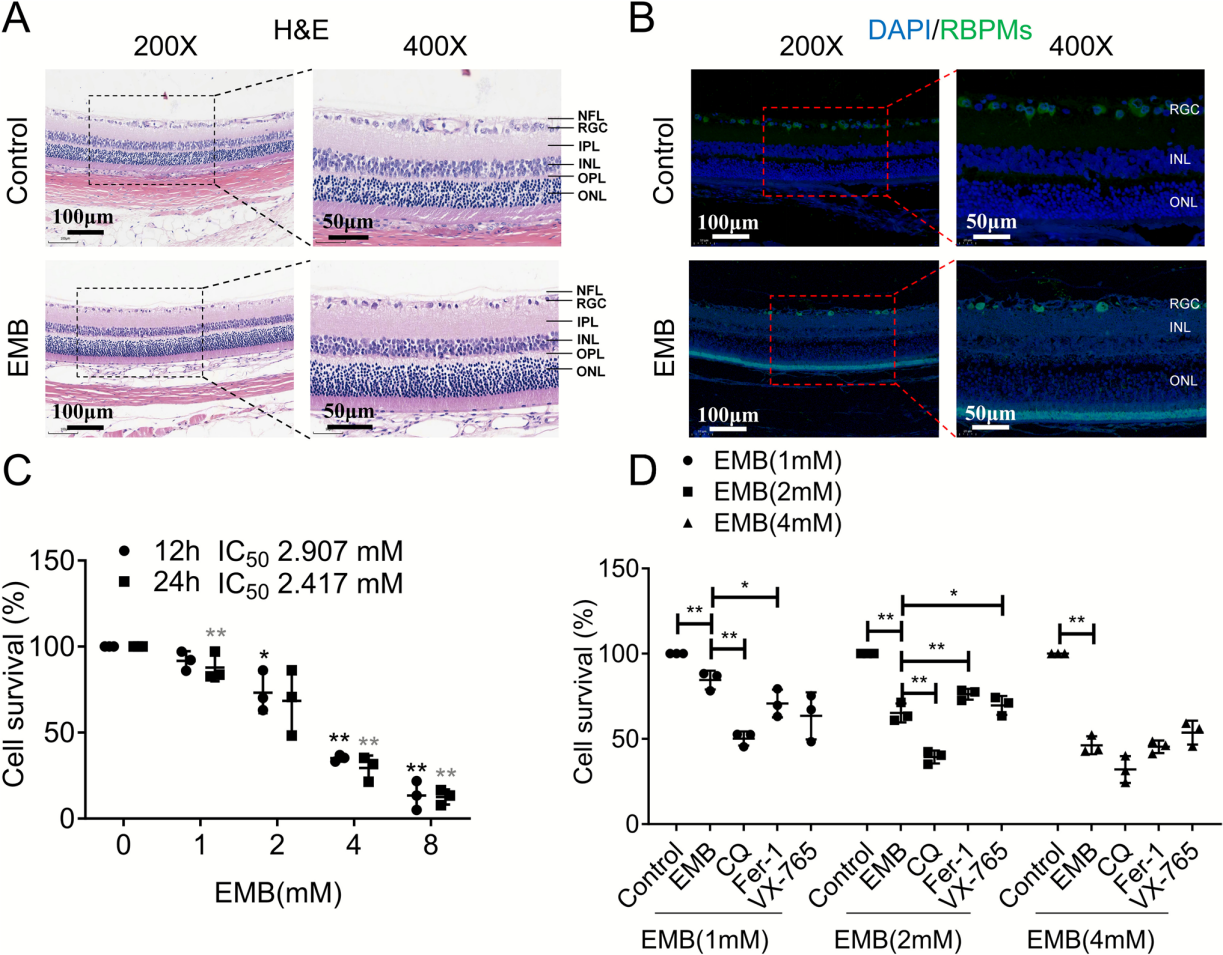
Effects of EMB on RGCs in EON rat model and RGC-5 cells. **A** Histological staining with H&E was performed on retinal sections obtained from control and EMB groups. Scale bar and magnification times: 100 μm and ×200 (left panel); 50 μm and ×400 (right panel). *NFL* nerve fiber layer, *RGC* retinal ganglion cell, *IPL* inner plexiform layer, *INL* inner nuclear layer, *OPL* outer plexiform layer, *ONL* outer nuclear layer. *N* = 3. **B** IF staining with RGC-specific marker antibodies (RBPMs) was conducted on retinas from control and EMB groups. Scale bar and magnification times: 100 μm and ×200 (left panel); 50 μm and ×400 (right panel). *RGC* retinal ganglion cell, *INL* inner nuclear layer, *ONL* outer nuclear layer. *N* = 3. **C** In vitro MTT assays using RGC-5 cells to assess EMB’s dose-dependent and time-dependent toxicity. The graph plots cell viability (%) against EMB concentration (0, 1, 2, 4, and 8 mM) in 12 or 24 h. The IC_50_ values represent the concentration of EMB required to inhibit cell viability by 50%. *p < 0.05, **p < 0.01. *N* = 3. **D** MTT assays were conducted with various cell death inhibitors (CQ: 20 μM; Fer-1: 2 μM; VX-765: 40 μM) to investigate the type of cell death induced by EMB (1 mM, 2 mM, and 4 mM) in RGC-5 cells. *N* = 3. *p < 0.05, **p < 0.01. Data shown are representative of three independent experimental replicates

Based on our previous study, structural damage to the p-RNFL and GCIPL induced by EMB treatment [12, 20], this study further explores the effects of EMB on an EON animal model and RGCs, aiming to elucidate the underlying mechanisms of EON. Wistar rats were used to create an EON model by administering EMB at 50 mg/kg. Histological staining with H&E showed cell loss and structural damage in the RGCs of EMB-treated rats (Fig. 1A). In addition, IF staining with RGCs marker antibodies (RBPMs) confirmed a significant decrease in RGCs and damage to the layer, corroborating impaired RGCs in EMB-administered rats (Fig. 1B). In vitro MTT assays using RGC-5 cells demonstrated EMB’s dose-dependent and time-dependent toxicity with an IC_50_ of 2.907 mM in 12 h and 2.417 mM in 24 h (Fig. 1C). Furthermore, to gain insights into the type of cell death induced by EMB, MTT assays were conducted with various cell death inhibitors: autophagy inhibitor (CQ) [21], ferroptosis inhibitor (Fer-1) [22], and pyroptosis inhibitor (VX-765) [23]. Among these, Fer-1 exhibited the strongest protective effect under 2 mM EMB treatment, with autophagy and pyroptosis playing lesser roles, indicating that ferroptosis might be the predominant mode of cell death in this context (Fig. 1D).

**Fig. 2:**
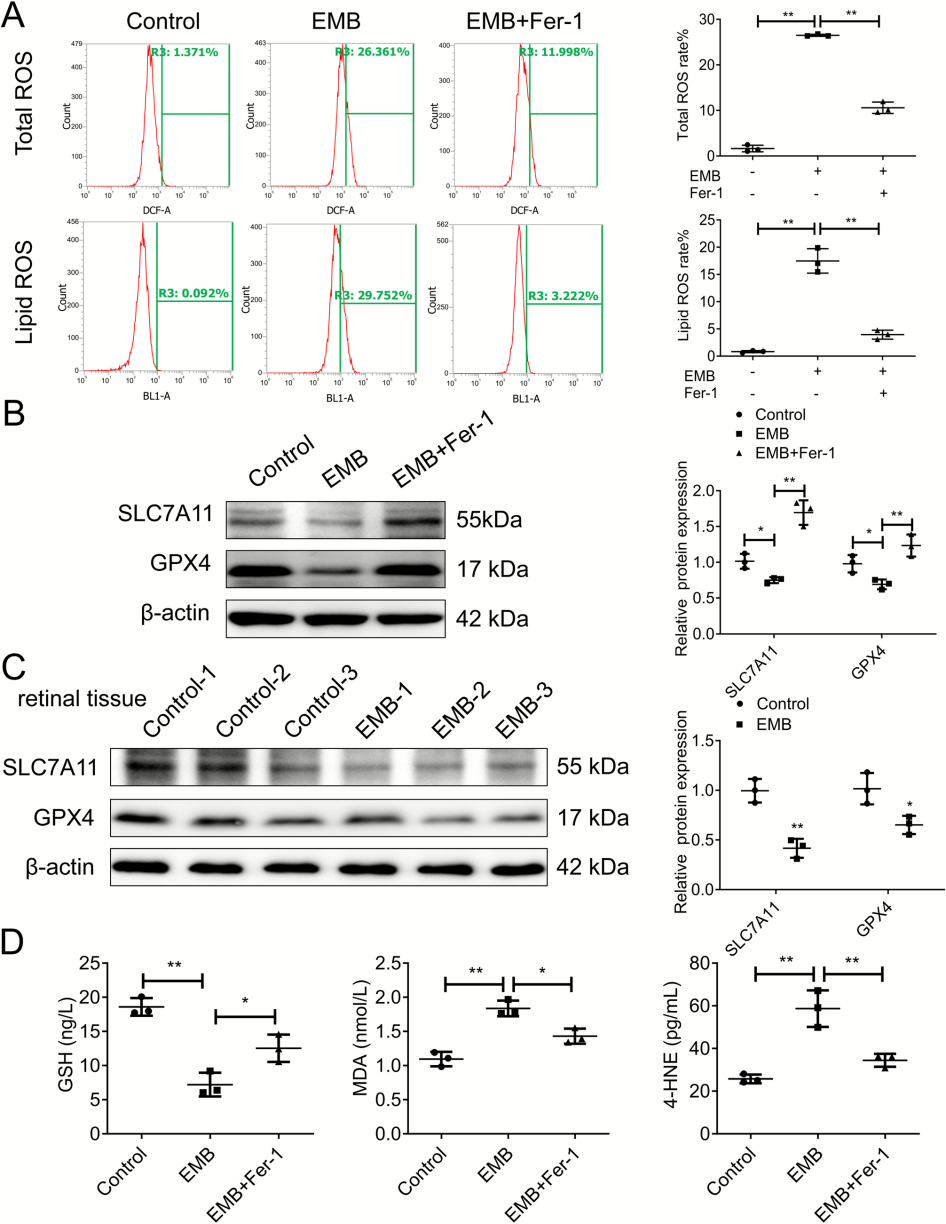
Molecular validation of ferroptosis in EMB-induced RGC-5 cell death. As the experimental group, RGC-5 cells in the logarithmic growth phase were treated with either 2 mM EMB alone or co-treated with 2 μM Fer-1 and 2 mM EMB for 24 h, with untreated RGC-5 cells serving as the control group for subsequent experiments. **A** Flow cytometry analyses were conducted to determine the ROS levels in each group. The histograms show the total ROS (upper panel) and lipid ROS (lower panel) in RGC-5 cells. *N* = 3. **p < 0.01. **B** WB analyses assessed the protein expression of SLC7A11 and GPX4 in each group. *N* = 3. *p < 0.05, **p < 0.01. **C** WB analyses were conducted to evaluate the protein expression of SLC7A11 and GPX4 in retinal tissue from the EON rats model, with no medication group as the control group. *N* = 3. *p < 0.05, **p < 0.01. **D** In each group, ELISA kit detection assays were conducted to measure the GSH, MDA, and 4-HNE levels, indicative of ferroptosis. *N* = 3. *p < 0.05, **p < 0.01. Data shown are representative of three independent experimental replicates

To validate ferroptosis in EMB-induced RGC death, we conducted further analyses focusing on key molecular events and markers. Flow cytometry demonstrated elevated total ROS and lipid ROS in EMB-treated RGC-5 cells, which was significantly reversed by Fer-1 (Fig. 2A). WB analysis showed downregulation of SLC7A11 and GPX4, critical antioxidants [24], in EMB-treated RGC-5 cells, with significant recovery upon Fer-1 co-treatment (Fig. 2B). It is worth mentioning that downregulation of SLC7A11 and GPX4 protein levels was also observed in retinal tissues of EON rat models (Fig. 2C). Elisa kit detection assays confirmed decreased GSH and increased MDA and 4-HNE levels in EMB-treated RGCs, indicative of ferroptosis [25], which were significantly reversed by co-treatment with Fer-1 (Fig. 2D). Taken together, our findings provide compelling evidence for the involvement of ferroptosis in EON.

### EMB damages RGCs by inhibiting SDHB mRNA and protein expression

**Fig. 3:**
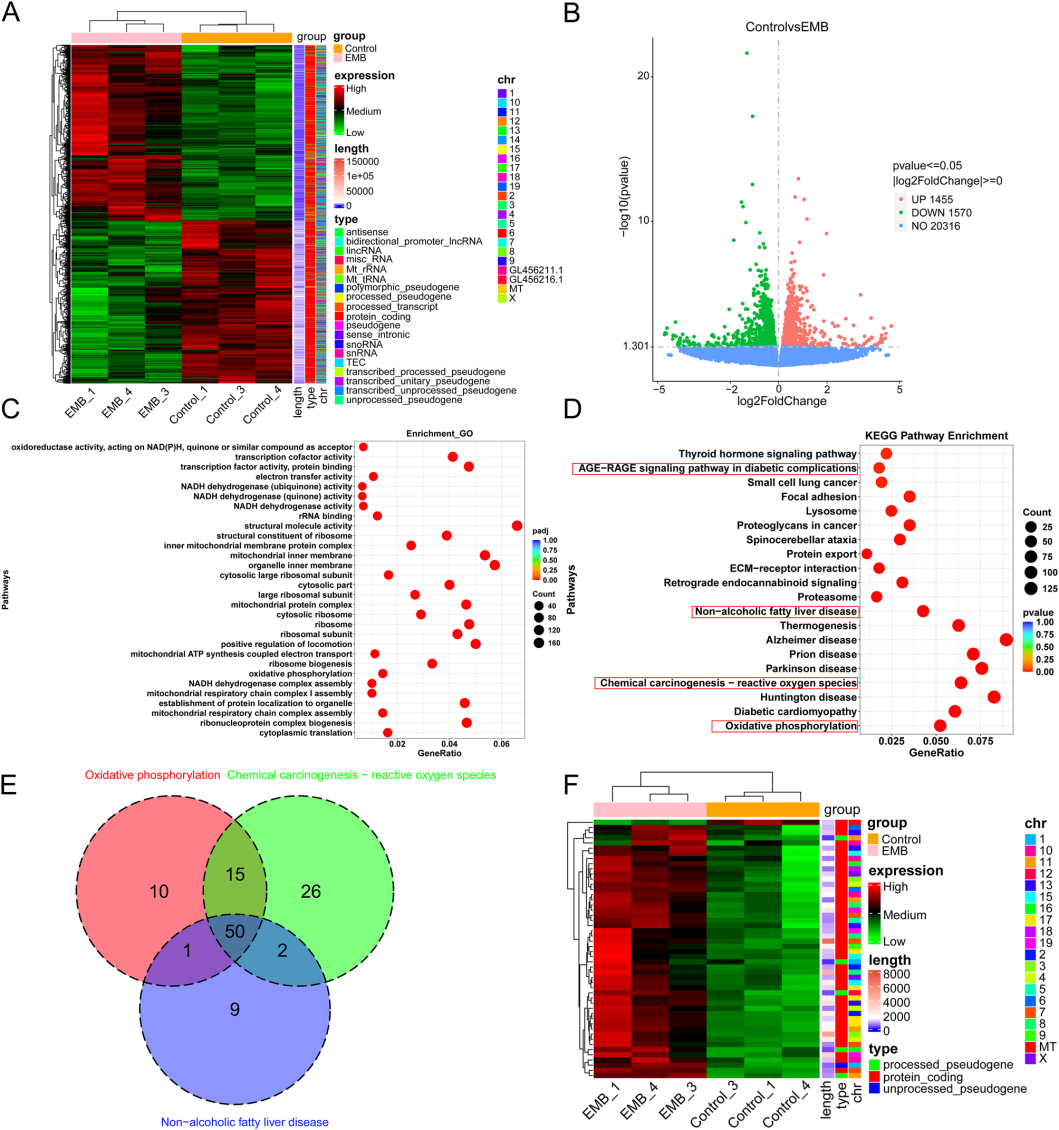
High-throughput sequencing analysis of DEGs in EMB-treated RGC-5 cells. RGC-5 cells in the logarithmic growth phase were treated with either 2 mM EMB or untreated to perform the high-throughput sequencing analysis. **A** Heatmap was used to visualize the DEGs in control vs. EMB-treated RGC-5 cells in sequencing data., showing hierarchical clustering of genes based on expression levels. The *x*-axis lists sample names, while the *y*-axis indicates the normalized FPKM values of DEGs. Redder colors indicate higher expression, greener lower. The heatmap also included: chromosome, gene length, and biological type. **B** Volcanic maps were used to visualize DEGs in control and EMB-treated RGC-5 cells. Up-regulated genes are represented by red dots and down-regulated genes by green dots. **C** GO enrichment analysis of DEGs and **D** KEGG analysis of altered pathways in EMB-treated RGC-5 cells, with bubble size showing gene count and color intensity indicating the statistical significance. **E** Venn diagram was used to identify the key genes intersected in the significantly altered pathways. **F** Focused heatmap analysis of overlapped genes identified in **E**. The “length” is the annotation of gene length, “type” is the annotation of gene type, and “chr” is the name of the chromosome, where the gene is located

To investigate EMB-induced molecular changes in RGCs, RGC-5 cells were treated with 2 mM EMB for 24 h, followed by total RNA extraction for high-throughput sequencing analysis. We created a heatmap and a volcano plot to visualize DEGs between control and EMB-treated groups. The heatmap showed a hierarchical clustering of genes, distinguishing high and low-expression genes due to EMB. The volcano plot identified genes with significant expression changes and statistical significance, hinting at key players in the EMB response with 1455 upregulated genes and 1570 downregulated genes (Fig. 3A, B). GO enrichment analysis revealed altered biological functions related to metabolism, stress response, and apoptotic regulation, suggesting EMB disrupts normal cellular functions (Fig. 3C). KEGG analysis identified significantly altered pathways, including oxidative phosphorylation, chemical carcinogenesis involving ROS, AGE–RAGE signaling pathway, and non-alcoholic fatty liver disease (NAFLD), which are critical for energy production, stress response, and metabolic homeostasis (Fig. 3D). Through intersection analysis of these key pathways, we identified six core genes (Cox6a2, Uqcrq, Cox5a, SDHB, Ndufb9, and Ndufa7) encoding essential subunits of mitochondrial complexes I, II, and IV (Fig. 3E) [26–31]. These genes were selected based on their established roles in maintaining electron transport chain integrity and their documented association with mitochondrial dysfunction [32], providing strong biological rationale for further investigation, visualized through focused heatmap analysis (Fig. 3F).

**Fig. 4:**
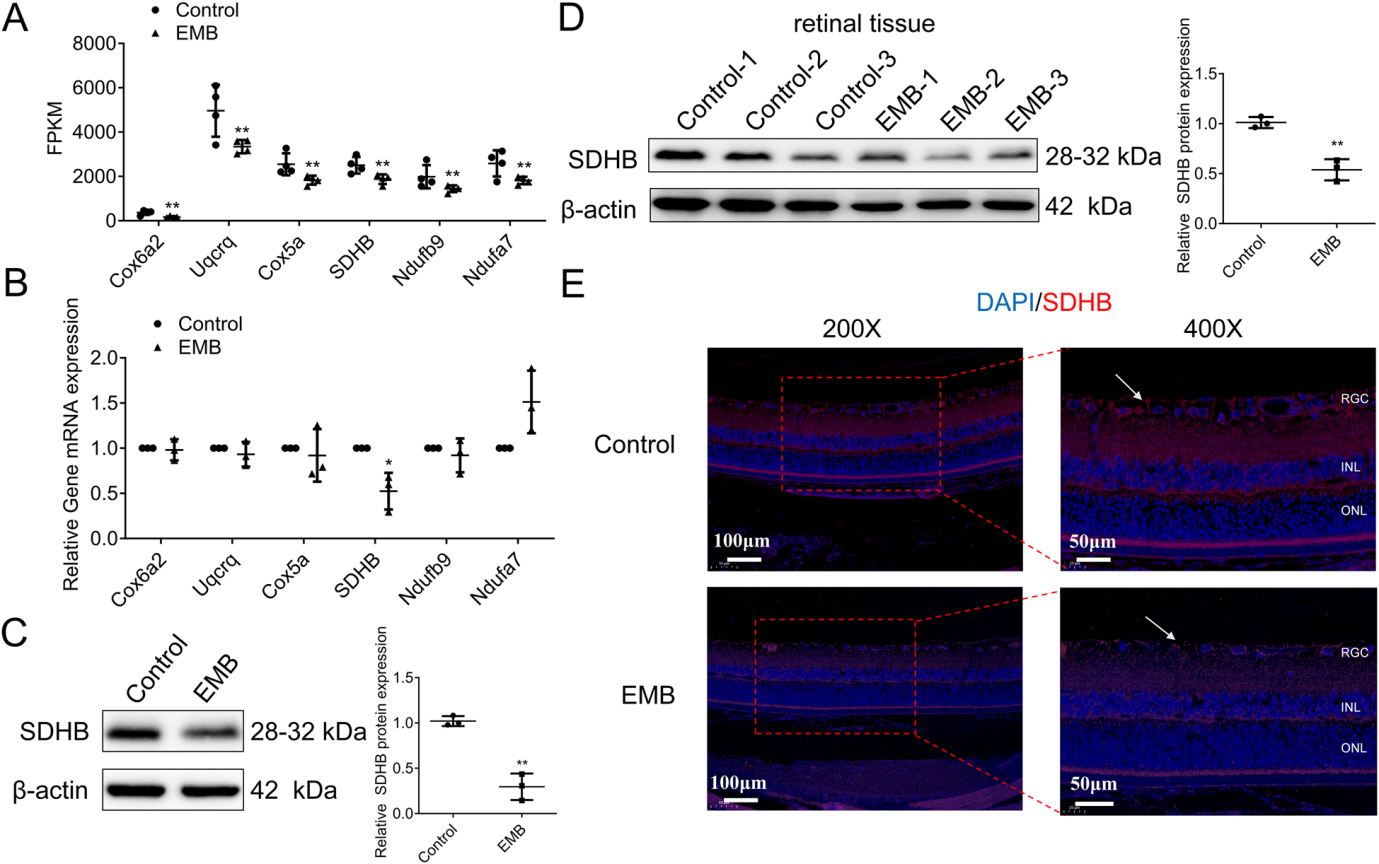
Assessment of key DEGs and SDHB expression in EMB-treated RGCs. **A** In sequencing data, FPKM values were analyzed for the intersection genes: Cox6a2, Uqcrq, Cox5a, SDHB, Ndufb9, and Ndufa7. *N* = 3. **p < 0.01. **B** RT-qPCR was used to validate the expression of these intersection genes in EMB-treated RGC-5 cells. *p < 0.05. **C, D** WB analyses were used to detect the SDHB protein levels in EMB-treated RGC-5 cells (**C**) and the retinal tissues from EON rats model (**D**). *N* = 3. **p < 0.01. **E** IF staining was conducted to assess SDHB expression in the retinal tissues of the EON rats model, with the nuclei counterstained with DAPI (blue fluorescence). Scale bar and magnification times: 100 μm and ×200 (left panel); 50 μm and ×400 (right panel). *RGC* retinal ganglion cell, *INL* inner nuclear layer, *ONL* outer nuclear layer. *N* = 3. Data shown are representative of three independent experimental replicates

We assessed FPKM values for these intersection genes in sequencing data, revealing EMB-induced downregulation of these critical respiratory chain components in RGCs compared to controls (Fig. 4A). Next, qPCR validation confirmed significant SDHB downregulation in EMB-treated RGCs (Fig. 4B), prompting subsequent focus on SDHB as a key responder to EMB toxicity. Furthermore, we also observed significantly decreased SDHB protein levels in EMB-treated RGCs and retinal tissues from EON rats (Fig. 4C, D). Moreover, IF staining revealed reduced SDHB expression in retinal ganglion cells of the EON rats mode (Fig. 4E), highlighting SDHB’s potential cell-specific roles in EMB-induced retinal health and disease.

### SDHB negatively regulates EMB-induced ferroptosis in RGCs

**Fig. 5:**
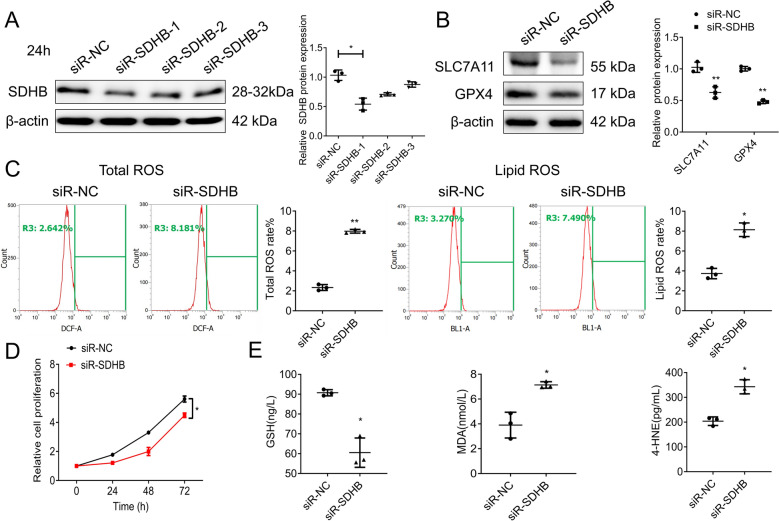
Effects of SDHB silencing on ROS levels, cell proliferation, and ferroptosis markers in RGC-5 cells. **A** WB assay was performed to confirm the silencing efficiency of SDHB expression in RGC-5 cells transfected with three specific siR-SDHB, compared to control cells transfected with a siR-NC. *N* = 3. *p < 0.05. **B** WB assays were used to detect expression levels of SLC7A11 and GPX4 in SDHB-silenced RGC-5 cells. *N* = 3. **p < 0.01. **C** Flow cytometry analyses were performed for ROS (left panel) and lipid ROS (right panel) in SDHB-silenced RGC-5 cells. *N* = 3. *p < 0.05, **p < 0.01. **D** Graph of MTT assay displayed the relative cell viability of siR-SDHB-treated RGC-5 cells compared to controls. *N* = 3. *p < 0.05. **E** Graphs of ELISA kit detection showed the relative levels of GSH, MDA, and 4-HNE in siR-SDHB-treated RGC-5 cells compared to controls. *N* = 3. *p < 0.05. Data shown are representative of three independent experimental replicates

**Fig. 6:**
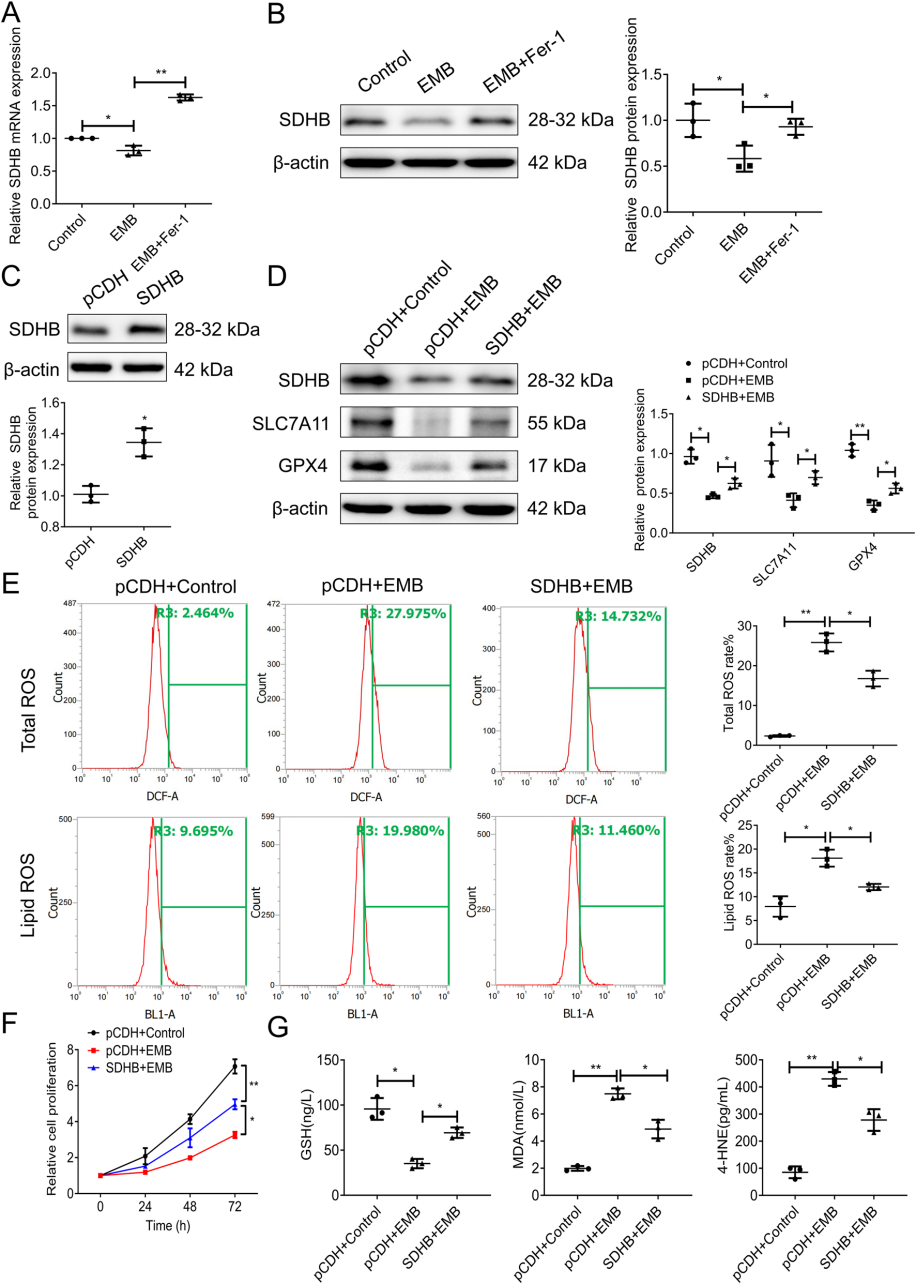
Recovery experiments demonstrating the regulatory role of SDHB in EMB-induced ferroptosis in RGC-5 cells. **A**, **B** RT-qPCR and WB assays were conducted to detect the effects of co-treating with 2 mM EMB and 2 μM Fer-1 on SDHB mRNA (**A**) and protein levels (**B**) in RGC-5 cells. *N* = 3. *p < 0.05 and **p < 0.01. **C** WB assay was performed to confirm the overexpression efficiency of SDHB in RGC-5 cells using an SDHB-overexpressed plasmid. *N* = 3. *p < 0.05. **D** Results of WB assays revealed the effects of treating RGC-5 cells with 2 mM EMB and transfecting them with overexpressed SDHB on SDHB, SLC7A11, and GPX4 protein levels. *N* = 3. *p < 0.05, **p < 0.01. **E** Flow cytometry analyses conducted to assess total ROS (upper panel) and lipid ROS (lower panel) levels in RGC-5 cells following co-treatment with 2 mM EMB and transfection with overexpressed SDHB. *N* = 3. *p < 0.05, **p < 0.01. **F** MTT assay was used to determine the relative cell proliferation of RGC-5 cells treated with 2 mM EMB and transfected with overexpressed SDHB. *N* = 3. *p < 0.05, **p < 0.01. **G** Elisa kit detection assays were employed to investigate the effects of treating RGC-5 cells with 2 mM EMB and overexpressing SDHB on the relative levels of ferroptosis biomarkers (GSH, MDA, and 4-HNE). *N* = 3. *p < 0.05, **p < 0.01. Data shown are representative of three independent experimental replicates

To further explore, we synthesized specific siRNA targeting SDHB to silence its expression (siR-SDHB) and confirmed its successful downregulation by WB assay. siR-SDHB-1 exhibited the highest silencing efficacy and was used in subsequent experiments (Fig. 5A). We then evaluated the expression of ferroptosis inhibitors SLC7A11 and GPX4, which decreased significantly in SDHB-silenced RGC-5 cells (Fig. 5B), suggesting disrupted antioxidant defenses and a predisposition to ferroptosis. Flow cytometry revealed increased total and lipid ROS levels in SDHB-silenced RGC-5 cells (Fig. 5C). Furthermore, MTT assays showed decreased cell proliferation in RGC-5 cells after silencing SDHB, further confirming SDHB depletion’s detrimental effects (Fig. 5D). Detection and quantification of GSH, MDA, and 4-HNE levels also supported the induction of ferroptosis in SDHB-silenced RGC-5 cells (Fig. 5E). Subsequently, the recovery experiment showed that while EMB decreased SDHB mRNA and protein levels in RGC-5 cells, co-treatment with Fer-1 significantly reversed this effect (Fig. 6A, B), suggesting a regulatory role of SDHB in EMB-induced ferroptosis. To further validate the functional role of SDHB in EMB-mediated ferroptosis, we constructed the SDHB-overexpressed plasmid and confirmed successful overexpression in RGC-5 cells by WB assay (Fig. 6C). Upon co-treatment with EMB and overexpressed SDHB plasmid in RGC-5 cells, we observed a partial reversal of the EMB-triggered reduction in SDHB, SLC7A11, and GPX4 protein levels (Fig. 6D). In addition, this recovery effect was also observed when total ROS levels and lipid ROS levels in co-treated cells were measured by flow cytometry (Fig. 6E), supporting SDHB’s weakening of EMB-induced ferroptosis. The result from the MTT assay revealed that the EMB-induced reduction in cell proliferation was somewhat restored in co-treated cells (Fig. 6F). Elisa kit detection assays also showed a corresponding change in the ferroptosis biomarkers level (Fig. 6G), which confirmed that SDHB had a certain protective response to EMB-induced ferroptosis of RGC-5 cells.

### EMB modulates SDHB promoter transcriptional activity by inhibiting Smad4–SDHB interaction in RGCs

**Fig. 7:**
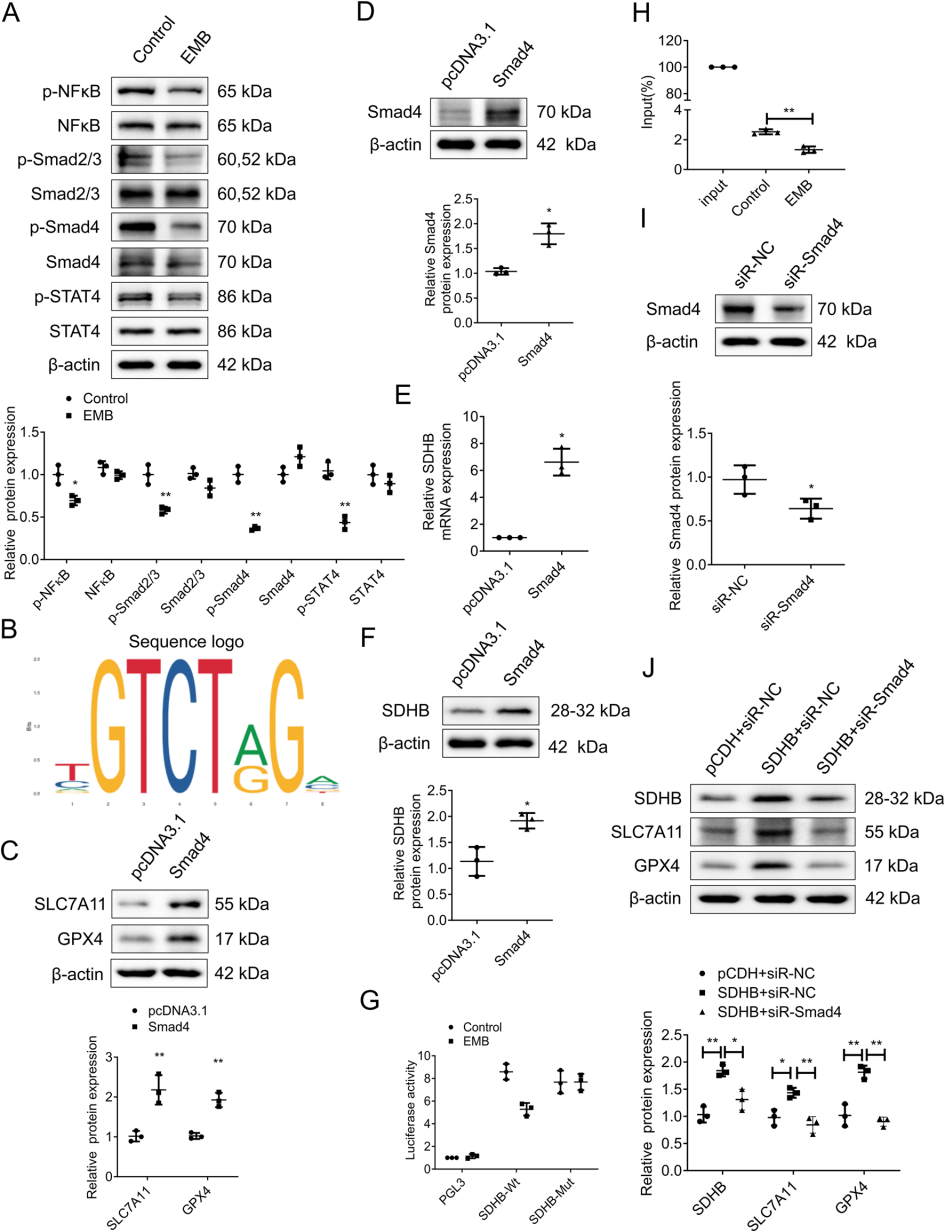
EMB modulates SDHB transcriptional activity via Smad4 in RGC-5 cells. **A** WB analyses were conducted to assess the NFκB, Smad2/3, Smad4, STAT4 levels, and their phosphorylation levels in RGC-5 cells treated with 2 mM EMB. *N* = 3. *p < 0.05, **p < 0.01. **B** Utilizing the JASPAR database, a prediction was made regarding the binding of Smad4 to the SDHB promoter. **C** Detection of SLC7A11 and GPX4 protein expression in Smad4-overexpressing RGC-5 cells by WB. *N* = 3. *p < 0.05. **D** WB assay was conducted to verify the Smad4 overexpression in RGC-5 cells. *N* = 3. *p < 0.05. **E**, **F** RT-qPCR and WB assays were performed to quantify SDHB mRNA (**E**) and protein (**F**) levels in Smad4-overexpressing RGC-5 cells. *N* = 3. *p < 0.05. **G** Dual-luciferase reporter assays were employed to demonstrate EMB’s regulation of SDHB promoter activity via Smad4. SDHB-Mut: mutating the Smad4 binding sites on SDHB promoters. *N* = 3. *p < 0.05. **H** CHIP assay to detect DNA enriched fragments of SDHB promoter was conducted using Smad4-specific antibodies in EMB-treated RGC-5 cells. *N* = 3. **p < 0.01. **I** WB analysis of Smad4 knockdown efficiency in RGC-5 cells. *N* = 3. *p < 0.05. **J** In SDHB-overexpressing RGC-5 cells with Smad4 knockdown, the protein expression levels of SDHB, SLC7A11, and GPX4 were detected by WB. Data shown are representative of three independent experimental replicates

Recent studies have emphasized the critical role of transcription factors in *Mycobacterium* tuberculosis resistance to EMB [33–36]. Based on prior research and our high-throughput sequencing data, we explored the EMB regulating SDHB transcriptional activity mechanisms, focusing on downstream transcription factors in the AGE–RAGE signaling pathway. WB analysis of RGC-5 cells treated with EMB revealed marked reductions in phosphorylated NFκB, Smad2/3, Smad4, and STAT4, hinting at EMB’s potential to modulate these transcription factors (Fig. 7A). Notably, Smad4 exhibited the greatest downregulation and was predicted to bind the SDHB promoter according to the JASPAR database (Fig. 7B), suggesting a regulatory role for Smad4 in SDHB expression. To further validate the functional consequences of Smad4 regulation, we performed Smad4 overexpression and assessed protein expression by WB analysis, which revealed significant upregulation of key ferroptosis-related proteins SLC7A11 and GPX4 (Fig. 7C), indicating Smad4's crucial role in maintaining cellular defense against ferroptosis. Upon Smad4 overexpression in RGC-5 cells (Fig. 7D), we observed a notable enhancement in SDHB mRNA and protein levels (Fig. 7E, F). Dual-luciferase reporter assays further confirmed EMB’s regulation of SDHB promoter activity via Smad4, with EMB-treated cells showing decreased luciferase activity. However, mutating the Smad4 binding sites on SDHB promoters abolished this effect (Fig. 7G). A CHIP assay with Smad4-specific antibodies in EMB-treated RGC-5 cells showed a significant reduction in Smad4 protein and SDHB promoter binding products, directly demonstrating EMB’s disruption of the Smad4–SDHB promoter interaction (Fig. 7H). To further validate the functional consequences of Smad4 regulation and its hierarchical position in the pathway, we performed critical rescue experiments. First, we confirmed efficient Smad4 knockdown by WB analysis (Fig. 7I). Subsequently, in SDHB-overexpressing RGC-5 cells, comparison between the SDHB + siR-NC group and the SDHB + siR-Smad4 group revealed that Smad4 knockdown significantly attenuated the enhanced protein levels of not only SDHB itself but also the key ferroptosis-related proteins SLC7A11 and GPX4 (Fig. 7J). This demonstrates that Smad4 operates upstream of SDHB and is indispensable for maintaining the expression of these crucial ferroptosis regulators. Together, these findings unequivocally establish Smad4's crucial role as an upstream transcriptional regulator in maintaining cellular defense against ferroptosis. These findings highlight that EMB regulates SDHB promoter transcriptional activity by inhibiting Smad4 in RGC-5 cells, at least partially.

## Discussion

This study presents compelling evidence for the mechanisms underlying EON, revealing that EMB triggers EON via ferroptosis-mediated pathways in RGCs and highlights the pivotal role of SDHB in this process. In addition, we demonstrate that EMB modulates SDHB promoter transcriptional activity by inhibiting the interaction between Smad4 and the SDHB promoter. The findings build upon our previous research that demonstrated structural damage to the p-RNFL and GCIPL following EMB treatment [12], and extend these observations by elucidating the molecular pathways involved in this neurotoxicity, offering new insights into the etiology of EMB-induced neurotoxicity.

While previous studies have demonstrated EMB's mitochondrial toxicity through zinc-mediated lysosomal permeabilization [37] and complex IV inhibition [38], the precise mechanism linking mitochondrial dysfunction to RGC death remained elusive. Our research bridges this gap by identifying ferroptosis as the crucial downstream effector. The connection between mitochondrial impairment and ferroptosis is well-established, as mitochondria serve as both the primary source and primary target of lipid peroxidation in this cell death pathway [39, 40]. Notably, our findings demonstrate that EMB-induced mitochondrial dysfunction specifically triggers the ferroptosis execution program through SDHB downregulation, representing a significant advancement in understanding EON pathogenesis.

Our transcriptomic analysis revealed that EMB predominantly affects genes involved in oxidative phosphorylation, with SDHB emerging as a central player. SDHB occupies a unique position in mitochondrial metabolism, functioning as both a TCA cycle enzyme and a component of the electron transport chain. This dual role makes it particularly vulnerable to metabolic stressors and crucial for maintaining redox homeostasis [41, 42]. Our experimental data confirm that SDHB deficiency disrupts mitochondrial function and creates a permissive environment for ferroptosis initiation, characterized by elevated lipid ROS and depletion of key antioxidant defenses (SLC7A11 and GPX4).

The discovery that Smad4 transcriptionally regulates SDHB represents a key mechanistic insight. Smad4, while best known for its role in TGF-β signaling, has recently been implicated in mitochondrial metabolism regulation [43, 44]. Our data demonstrate that EMB-mediated Smad4 inhibition disrupts SDHB transcription, thereby compromising mitochondrial integrity and activating ferroptosis. This Smad4–SDHB–ferroptosis axis provides a unified framework connecting upstream EMB exposure to downstream RGC degeneration.

The convergence of mitochondrial dysfunction and ferroptosis in EON has important therapeutic implications. Our findings that both ferroptosis inhibitors and SDHB overexpression confer protection suggest multiple intervention points. Furthermore, the observed partial efficacy of these interventions indicates that parallel pathways, including pyroptosis, likely contribute to RGC death. This complexity mirrors findings in other neurodegenerative conditions, where multiple cell death pathways operate concurrently [45–47].

Our findings indicate that pyroptosis may contribute to EMB-induced RGC death alongside ferroptosis. This is supported by the significant neuroprotection observed with caspase-1 inhibitor VX-765 at 2 mM EMB concentration (Fig. 1D). Pyroptosis is an inflammatory cell death pathway mediated by caspase-1 cleavage of Gasdermin D (GSDMD), leading to membrane pore formation and release of inflammatory cytokines IL-1β and IL-18 [48]. This mechanism aligns with established evidence of pyroptosis in neuronal damage. In neurological disorders, pyroptosis has been identified as a central process contributing to neuronal damage. For instance, in models of Alzheimer's and Parkinson's diseases, pathological proteins such as β-amyloid and α-synuclein can activate inflammasomes and trigger the caspase-1/GSDMD pathway, ultimately resulting in neuronal death [49]. More importantly, studies in glaucoma and optic nerve injury models have similarly demonstrated GSDMD-mediated pyroptosis in RGCs [50, 51]. Our findings align with these reports, suggesting that EMB, as an exogenous stressor, may activate inflammasomes (e.g., NLRP3) within RGCs, initiating the canonical pyroptosis pathway. Notably, crosstalk may exist between cell death pathways. Ferroptosis-derived lipid peroxidation products can activate inflammasomes [52], potentially explaining the concurrent activation of both pathways at 2 mM EMB. This suggests EMB-induced ferroptosis might amplify pyroptosis through inflammatory signaling, resulting in enhanced RGC loss.

This study has several limitations that should be acknowledged. While the 50 mg/kg/day dosage of EMB utilized in this study exceeds standard human-equivalent dosing, it was selected based on established preclinical protocols that have consistently demonstrated efficacy in inducing characteristic retinal pathology in rodent models [53, 54]. This approach was necessary to ensure robust phenotypic expression for mechanistic investigation. It is important to acknowledge that our findings primarily elucidate pathological mechanisms rather than define a clinical dosing regimen. Future studies are warranted to establish a therapeutic window and optimize dosage for potential translational applications, which represents an essential next step toward clinical relevance. In addition, it should be noted that the RGC-5 cell line employed in this study has been reclassified as a rat retinal precursor cell line rather than a pure retinal ganglion cell population [16]. This updated classification warrants careful interpretation of our findings. Nevertheless, this cell line remains a valuable model in visual system disease research, particularly for exploring neuroprotective pathways and drug screening. Furthermore, while primary RGC cultures might provide more direct evidence, considering the technical challenges and viability limitations of primary cultures, the RGC-5 model offers a reliable platform for our initial investigation into EMB's neuroprotective mechanisms. We propose that these findings should be considered as preliminary mechanistic evidence in a retinal neuronal model, and we plan to further validate these pathways using conditional knockout animal models in future studies. Moreover, we acknowledge that direct in vivo validation through genetic manipulation of Smad4 or SDHB remains to be established. Future studies utilizing conditional knockout models or viral-mediated gene delivery in the retina would be valuable to confirm this molecular pathway in an intact physiological system. This represents an important direction for further investigation.

## Conclusion

This study presents novel findings on the mechanisms of EMB-induced neurotoxicity (specific to EON), highlighting the role of SDHB and ferroptosis in this process. Specifically, SDHB promoter activity is modulated through EMB inhibition of Smad4 expression to participate in EMB-induced ferroptosis of RGCs.

## Supplementary Information

Below is the link to the electronic supplementary material.

Supplementary file1 ControlvsEMB.all_GO (PDF 128 KB)

Supplementary file2 ControlvsEMB.all_KEGG (PDF 141 KB)

Supplementary file3 ControlvsEMB_volcano (PDF 12843 KB)

Supplementary file4 Heatmap (PDF 1166 KB)

Supplementary file5 Venn (PDF 123 KB)

Supplementary file6 Venn_heatmap (PDF 185 KB)

Supplementary file7 Western blot original data (PDF 354 KB)

## Data Availability

The data sets generated during and analyzed during the current study are available from the corresponding author on reasonable request.

## References

[1] Wang T, Jiao WW, Shen AD. Progress on mechanism of ethambutol resistance in *Mycobacterium tuberculosis*. Yi Chuan. 2016;38:910–7. 27806932 10.16288/j.yczz.16-111

[2] Srivastava S, Ayyagari A, Dhole TN, Nyati KK, Dwivedi SK. *Emb* nucleotide polymorphisms and the role of embB306 mutations in *Mycobacterium tuberculosis* resistance to ethambutol. Int J Med Microbiol. 2009;299:269–80. 19010731 10.1016/j.ijmm.2008.07.001

[3] Zhang XH, Xie Y, Xu QG, et al. Mitochondrial mutations in ethambutol-induced optic neuropathy. Front Cell Dev Biol. 2021;9:754676. 34676220 10.3389/fcell.2021.754676 PMC8525703

[4] Yang HK, Park MJ, Lee JH, Lee CT, Park JS, Hwang JM. Incidence of toxic optic neuropathy with low-dose ethambutol. Int J Tuberc Lung Dis. 2016;20:261–4. 26792482 10.5588/ijtld.15.0275

[5] Chamberlain PD, Sadaka A, Berry S, Lee AG. Ethambutol optic neuropathy. Curr Opin Ophthalmol. 2017;28:545–51. 28759559 10.1097/ICU.0000000000000416

[6] Grzybowski A, Zülsdorff M, Wilhelm H, Tonagel F. Toxic optic neuropathies: an updated review. Acta Ophthalmol. 2015;93:402–10. 25159832 10.1111/aos.12515

[7] Chen SC, Lin MC, Sheu SJ. Incidence and prognostic factor of ethambutol-related optic neuropathy: 10-year experience in southern Taiwan. Kaohsiung J Med Sci. 2015;31:358–62. 26162816 10.1016/j.kjms.2015.05.004 PMC11916502

[8] Lee EJ, Kim SJ, Choung HK, Kim JH, Yu YS. Incidence and clinical features of ethambutol-induced optic neuropathy in Korea. J Neuroophthalmol. 2008;28:269–77. 19145123 10.1097/WNO.0b013e31818e3c6b

[9] Lee JY, Han J, Seo JG, Park KA, Oh SY. Diagnostic value of ganglion cell-inner plexiform layer for early detection of ethambutol-induced optic neuropathy. Br J Ophthalmol. 2019;103:379–84. 29699978 10.1136/bjophthalmol-2018-312063

[10] Chaitanuwong P, Srithawatpong S, Ruamviboonsuk P, Apinyawasisuk S, Watcharapanjamart A, Moss HE. Incidence, risk factors and ophthalmic clinical characteristic of ethambutol-induced optic neuropathy: 7-year experience. Front Ophthalmol. 2023;3:1152215. 10.3389/fopht.2023.1152215 PMC11182281 38983080

[11] Chai SJ, Foroozan R. Decreased retinal nerve fibre layer thickness detected by optical coherence tomography in patients with ethambutol-induced optic neuropathy. Br J Ophthalmol. 2007;91:895–7. 17215265 10.1136/bjo.2006.113118 PMC1955652

[12] Su L, Li Q, Zhu L, et al. Characterisation of macular superficial vessel density alteration in preclinical ethambutol-induced optic neuropathy using optical coherence tomography angiography. Br J Ophthalmol. 2022;106:422–6. 33243831 10.1136/bjophthalmol-2020-317742

[13] Cinici E, Cetin N, Ahiskali I, et al. The effect of thiamine pyrophosphate on ethambutol-induced ocular toxicity. Cutan Ocul Toxicol. 2016;35:222–7. 26339826 10.3109/15569527.2015.1077857

[14] Huang SP, Chien JY, Tsai RK. Ethambutol induces impaired autophagic flux and apoptosis in the rat retina. Dis Model Mech. 2015;8:977–87. 26092127 10.1242/dmm.019737 PMC4527287

[15] Sanz-Morello B, Ahmadi H, Vohra R, et al. Oxidative stress in optic neuropathies. Antioxidants. 2021. 10.3390/antiox10101538. 34679672 10.3390/antiox10101538 PMC8532958

[16] Krishnamoorthy RR, Clark AF, Daudt D, Vishwanatha JK, Yorio T. A forensic path to RGC-5 cell line identification: lessons learned. Invest Ophthalmol Vis Sci. 2013;54:5712–9. 23975727 10.1167/iovs.13-12085

[17] Van Bergen NJ, Wood JP, Chidlow G, et al. Recharacterization of the RGC-5 retinal ganglion cell line. Invest Ophthalmol Vis Sci. 2009;50:4267–72. 19443730 10.1167/iovs.09-3484

[18] Nusanti S, Sari RI, Siregar NC, Sidik M. The effect of Citicoline on Ethambutol Optic Neuropathy: histopathology and immunohistochemistry analysis of retina ganglion cell damage level in rat model. J Ocul Pharmacol Ther. 2022;38:584–9. 36074092 10.1089/jop.2022.0005

[19] Li G, Luo Y, Zhang Q, et al. The RBPMS(CreERT2-tdTomato) mouse line for studying retinal and vascular relevant diseases. iScience. 2023;26:108111. 37867934 10.1016/j.isci.2023.108111 PMC10589894

[20] Sheng WY, Su LY, Ge W, Wu SQ, Zhu LW. Analysis of structural injury patterns in peripapillary retinal nerve fibre layer and retinal ganglion cell layer in ethambutol-induced optic neuropathy. BMC Ophthalmol. 2021;21:132. 33691649 10.1186/s12886-021-01881-y PMC7945056

[21] Liang DH, Choi DS, Ensor JE, Kaipparettu BA, Bass BL, Chang JC. The autophagy inhibitor chloroquine targets cancer stem cells in triple negative breast cancer by inducing mitochondrial damage and impairing DNA break repair. Cancer Lett. 2016;376:249–58. 27060208 10.1016/j.canlet.2016.04.002 PMC4864217

[22] Bai T, Li M, Liu Y, Qiao Z, Wang Z. Inhibition of ferroptosis alleviates atherosclerosis through attenuating lipid peroxidation and endothelial dysfunction in mouse aortic endothelial cell. Free Radic Biol Med. 2020;160:92–102. 32768568 10.1016/j.freeradbiomed.2020.07.026

[23] Tao H, Zhao H, Mo A, et al. VX-765 attenuates silica-induced lung inflammatory injury and fibrosis by modulating alveolar macrophages pyroptosis in mice. Ecotoxicol Environ Saf. 2023;249:114359. 36508797 10.1016/j.ecoenv.2022.114359

[24] Chen X, Li J, Kang R, Klionsky DJ, Tang D. Ferroptosis: machinery and regulation. Autophagy. 2021;17:2054–81. 32804006 10.1080/15548627.2020.1810918 PMC8496712

[25] Ye T, Yang W, Gao T, et al. Trastuzumab-induced cardiomyopathy via ferroptosis-mediated mitochondrial dysfunction. Free Radic Biol Med. 2023;206:143–61. 37392951 10.1016/j.freeradbiomed.2023.06.019

[26] Shi W, He L, Li R, Cao J. Role of mitochondrial complex I genes in host plant expansion of Bactrocera tau (Tephritidae: Diptera) by CRISPR/Cas9 system. Insect Sci. 2025. 10.1111/1744-7917.13495. 39829059 10.1111/1744-7917.13495 PMC12905475

[27] Jiang M, Song Y, Chen X, et al. COX6A2 deficiency leads to cardiac remodeling in human pluripotent stem cell-derived cardiomyocytes. Stem Cell Res Ther. 2023;14:357. 38072986 10.1186/s13287-023-03596-x PMC10712066

[28] Gu J, Wu M, Guo R, et al. The architecture of the mammalian respirasome. Nature. 2016;537:639–43. 27654917 10.1038/nature19359

[29] Barel O, Shorer Z, Flusser H, et al. Mitochondrial complex III deficiency associated with a homozygous mutation in UQCRQ. Am J Hum Genet. 2008;82:1211–6. 18439546 10.1016/j.ajhg.2008.03.020 PMC2427202

[30] Baertling F, Al-Murshedi F, Sanchez-Caballero L, et al. Mutation in mitochondrial complex IV subunit COX5A causes pulmonary arterial hypertension, lactic acidemia, and failure to thrive. Hum Mutat. 2017;38:692–703. 28247525 10.1002/humu.23210

[31] Astuti D, Latif F, Dallol A, et al. Gene mutations in the succinate dehydrogenase subunit SDHB cause susceptibility to familial pheochromocytoma and to familial paraganglioma. Am J Hum Genet. 2001;69:49–54. 11404820 10.1086/321282 PMC1226047

[32] Javadov S, Jang S, Chapa-Dubocq XR, Khuchua Z, Camara AK. Mitochondrial respiratory supercomplexes in mammalian cells: structural versus functional role. J Mol Med (Berl). 2021;99:57–73. 33201259 10.1007/s00109-020-02004-8 PMC7785696

[33] Li JJ, Wu SF, Bai FX. Action mechanism of Ethambutol Tablets on Pulmonary Tuberculosis Rat Model Based on Janus Kinase/Signal Transducer and Activator of Transcription Signaling Pathway. Zhongguo Yi Xue Ke Xue Yuan Xue Bao. 2022;44:555–62. 36065686 10.3881/j.issn.1000-503X.14923

[34] Liou RH, Chen SW, Cheng HC, et al. The efficient induction of human retinal ganglion-like cells provides a platform for studying optic neuropathies. Cell Mol Life Sci. 2023;80:239. 37540379 10.1007/s00018-023-04890-w PMC10403410

[35] Colangeli R, Helb D, Vilchèze C, et al. Transcriptional regulation of multi-drug tolerance and antibiotic-induced responses by the histone-like protein Lsr2 in *M. tuberculosis*. PLoS Pathog. 2007;3:e87. 17590082 10.1371/journal.ppat.0030087 PMC1894825

[36] Sharma K, Gupta M, Krupa A, Srinivasan N, Singh Y. EmbR, a regulatory protein with ATPase activity, is a substrate of multiple serine/threonine kinases and phosphatase in Mycobacterium tuberculosis. FEBS J. 2006;273:2711–21. 16817899 10.1111/j.1742-4658.2006.05289.x

[37] Chung H, Yoon YH, Hwang JJ, Cho KS, Koh JY, Kim JG. Ethambutol-induced toxicity is mediated by zinc and lysosomal membrane permeabilization in cultured retinal cells. Toxicol Appl Pharmacol. 2009;235:163–70. 19063910 10.1016/j.taap.2008.11.006

[38] Guillet V, Chevrollier A, Cassereau J, et al. Ethambutol-induced optic neuropathy linked to OPA1 mutation and mitochondrial toxicity. Mitochondrion. 2010;10:115–24. 19900585 10.1016/j.mito.2009.11.004

[39] Gan B. Mitochondrial regulation of ferroptosis. J Cell Biol. 2021. 10.1083/jcb.202105043. 34328510 10.1083/jcb.202105043 PMC8329737

[40] Dixon SJ, Olzmann JA. The cell biology of ferroptosis. Nat Rev Mol Cell Biol. 2024;25:424–42. 38366038 10.1038/s41580-024-00703-5 PMC12187608

[41] Goncalves J, Moog S, Morin A, et al. Loss of SDHB promotes dysregulated iron homeostasis, oxidative stress, and sensitivity to ascorbate. Cancer Res. 2021;81:3480–94. 34127497 10.1158/0008-5472.CAN-20-2936 PMC7616967

[42] Hwang MS, Rohlena J, Dong LF, Neuzil J, Grimm S. Powerhouse down: complex II dissociation in the respiratory chain. Mitochondrion. 2014;19 Pt A:20–8. 24933571 10.1016/j.mito.2014.06.001

[43] Li J, Sun YBY, Chen W, et al. Smad4 promotes diabetic nephropathy by modulating glycolysis and OXPHOS. EMBO Rep. 2020;21:e48781. 31916354 10.15252/embr.201948781 PMC7001498

[44] Wang H, Chen Y, Wu G. SDHB deficiency promotes TGFbeta-mediated invasion and metastasis of colorectal cancer through transcriptional repression complex SNAIL1-SMAD3/4. Transl Oncol. 2016;9:512–20. 27816688 10.1016/j.tranon.2016.09.009 PMC5097976

[45] Zhao N, Li S, Wu H, et al. Ferroptosis: an energetic villain of age-related macular degeneration. Biomedicines. 2025. 10.3390/biomedicines13040986. 40299661 10.3390/biomedicines13040986 PMC12024642

[46] Tian HY, Huang BY, Nie HF, et al. The interplay between mitochondrial dysfunction and ferroptosis during ischemia-associated central nervous system diseases. Brain Sci. 2023. 10.3390/brainsci13101367. 37891735 10.3390/brainsci13101367 PMC10605666

[47] Zhou M, Xu K, Ge J, et al. Targeting ferroptosis in Parkinson’s disease: mechanisms and emerging therapeutic strategies. Int J Mol Sci. 2024. 10.3390/ijms252313042. 39684753 10.3390/ijms252313042 PMC11641825

[48] Shi J, Zhao Y, Wang K, et al. Cleavage of GSDMD by inflammatory caspases determines pyroptotic cell death. Nature. 2015;526:660–5. 26375003 10.1038/nature15514

[49] Heneka MT, Kummer MP, Stutz A, et al. NLRP3 is activated in Alzheimer’s disease and contributes to pathology in APP/PS1 mice. Nature. 2013;493:674–8. 23254930 10.1038/nature11729 PMC3812809

[50] Lv W, Wu X, Dou Y, et al. Homer1 protects against retinal ganglion cell pyroptosis by inhibiting endoplasmic reticulum stress-associated TXNIP/NLRP3 inflammasome activation after middle cerebral artery occlusion-induced retinal ischemia. Int J Mol Sci. 2023. 10.3390/ijms242316811. 38069134 10.3390/ijms242316811 PMC10706256

[51] Chen H, Deng Y, Gan X, et al. NLRP12 collaborates with NLRP3 and NLRC4 to promote pyroptosis inducing ganglion cell death of acute glaucoma. Mol Neurodegener. 2020;15:26. 32295623 10.1186/s13024-020-00372-w PMC7161290

[52] Park M, Park S, Choi Y, et al. The mechanism underlying correlation of particulate matter-induced ferroptosis with inflammasome activation and iron accumulation in macrophages. Cell Death Discov. 2024;10:144. 38491062 10.1038/s41420-024-01874-y PMC10943117

[53] Karakurt Y, Suleyman H, Keskin Cimen F, et al. The effects of lutein on optic nerve injury induced by ethambutol and isoniazid: an experimental study. Cutan Ocul Toxicol. 2019;38:136–40. 30362367 10.1080/15569527.2018.1539010

[54] Sahin A, Kursat Cingu A, Kaya S, et al. The protective effects of caffeic acid phenethyl ester in isoniazid and ethambutol-induced ocular toxicity of rats. Cutan Ocul Toxicol. 2013;32:228–33. 23351037 10.3109/15569527.2012.759958

