# Supplementary figures and images for: Ethambutol induces optic neuropathy through SDHB-mediated ferroptosis in retinal ganglion cells via Smad4 pathway
Source: Hum Cell. 2026 Jan 21;39(2):37. doi: 10.1007/s13577-025-01342-4 (PMC12823716; doi:10.1007/s13577-025-01342-4)

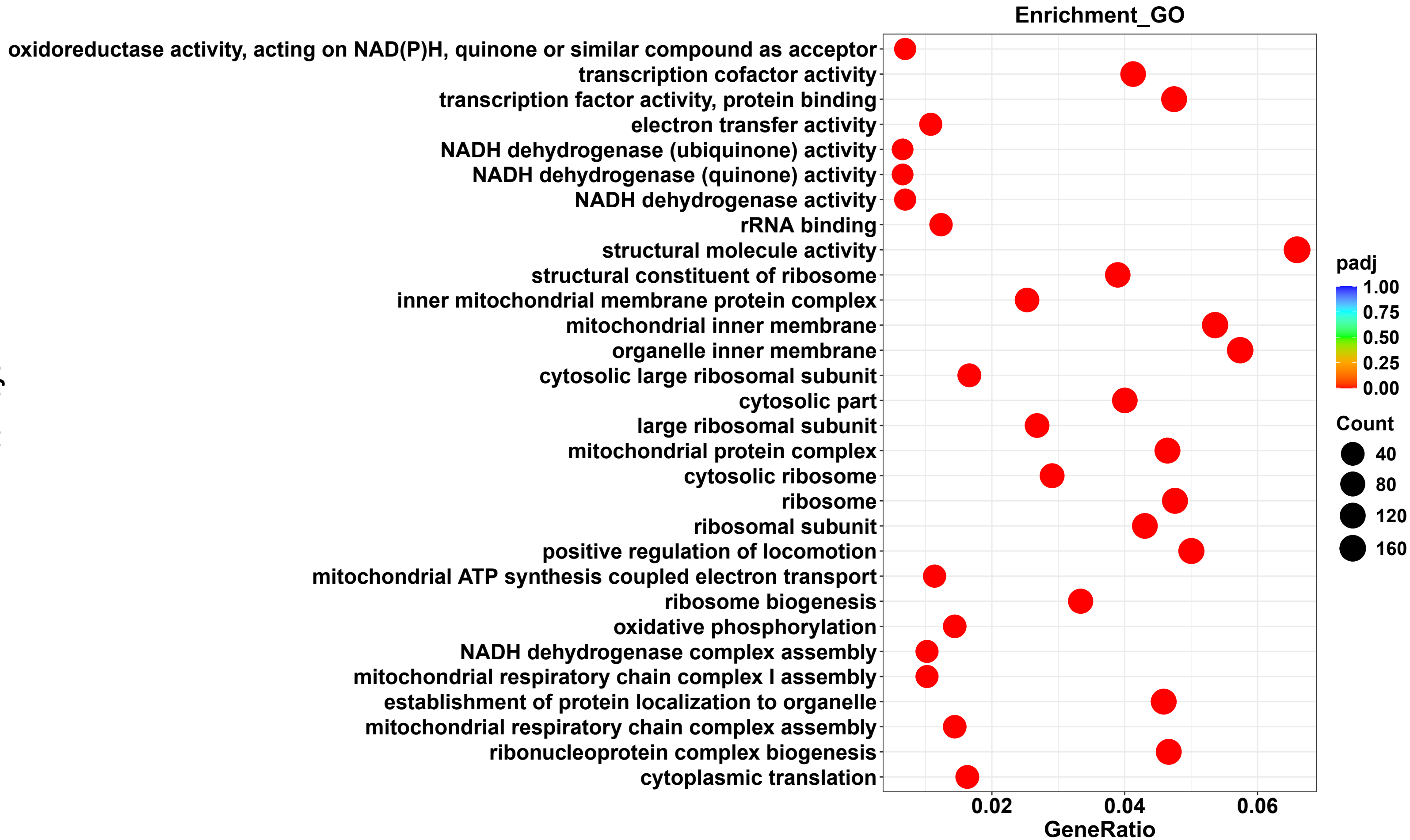

Supplement: Supplementary file 1 — Supplementary file1 ControlvsEMB.all_GO (PDF 128 KB) [file 13577_2025_1342_MOESM1_ESM.pdf]

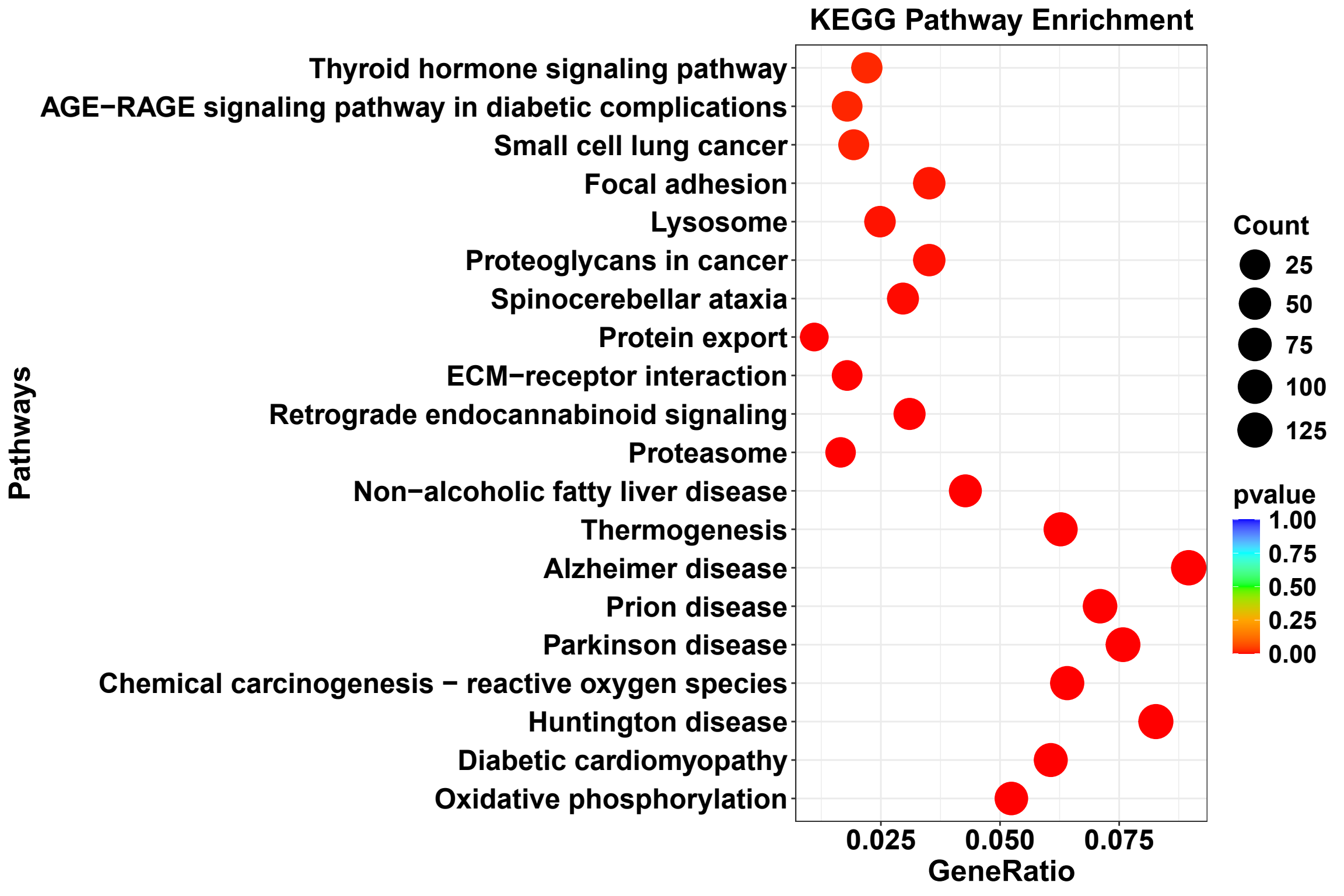

Supplement: Supplementary file 2 — Supplementary file2 ControlvsEMB.all_KEGG (PDF 141 KB) [file 13577_2025_1342_MOESM2_ESM.pdf]

# ControlvsEMB

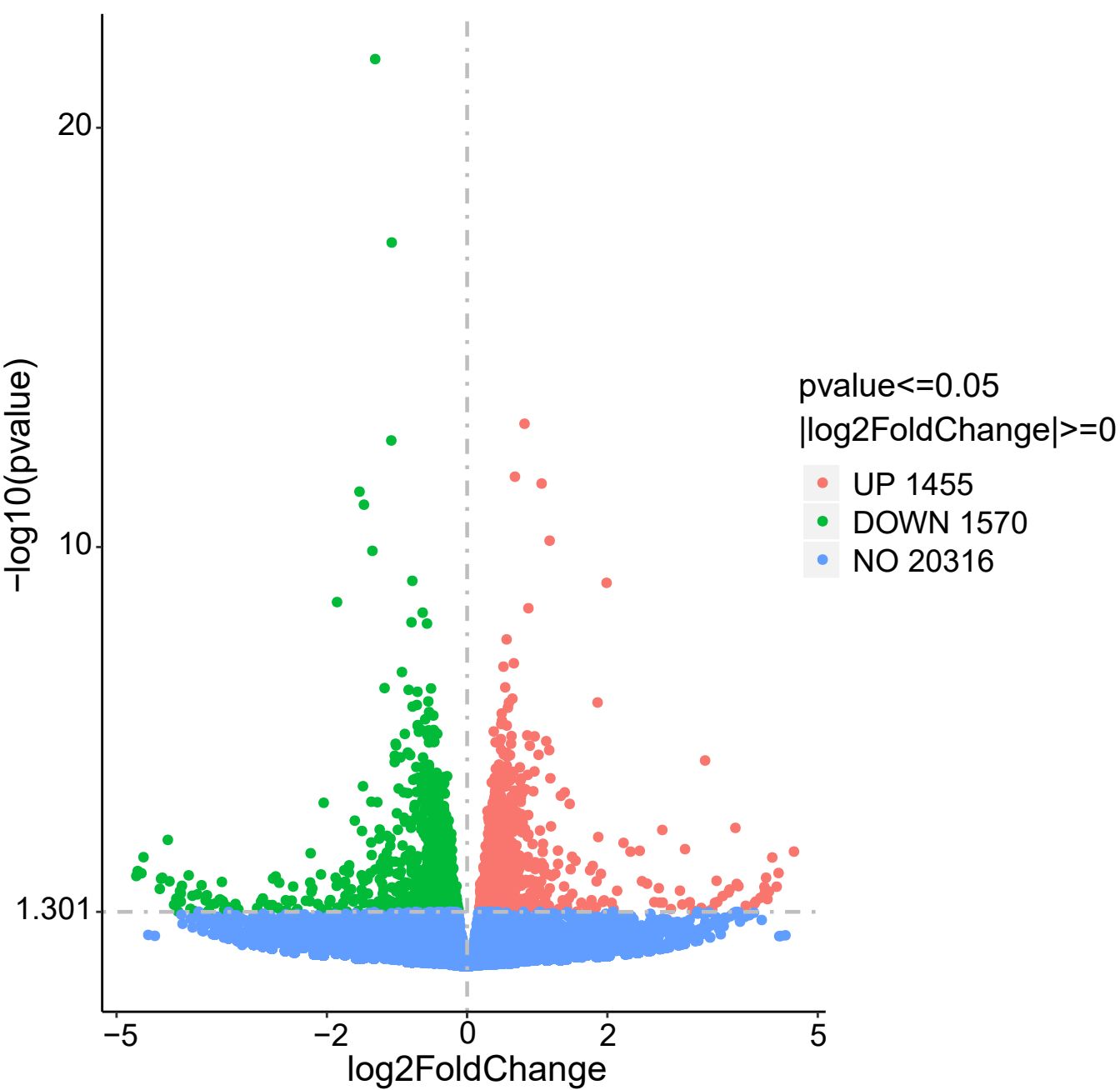

Supplement: Supplementary file 3 — Supplementary file3 ControlvsEMB_volcano (PDF 12843 KB) [file 13577_2025_1342_MOESM3_ESM.pdf]

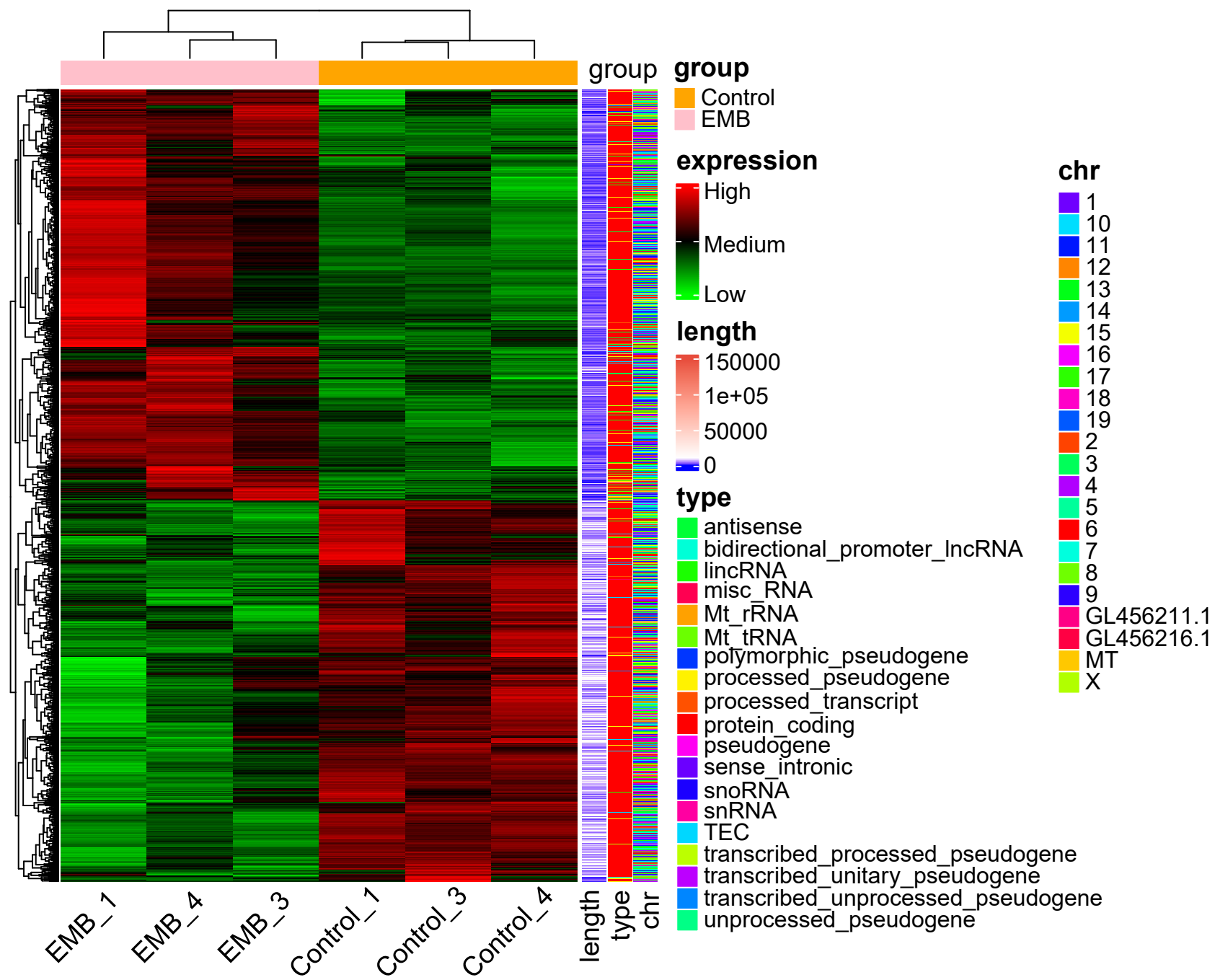

Supplement: Supplementary file 4 — Supplementary file4 Heatmap (PDF 1166 KB) [file 13577_2025_1342_MOESM4_ESM.pdf]

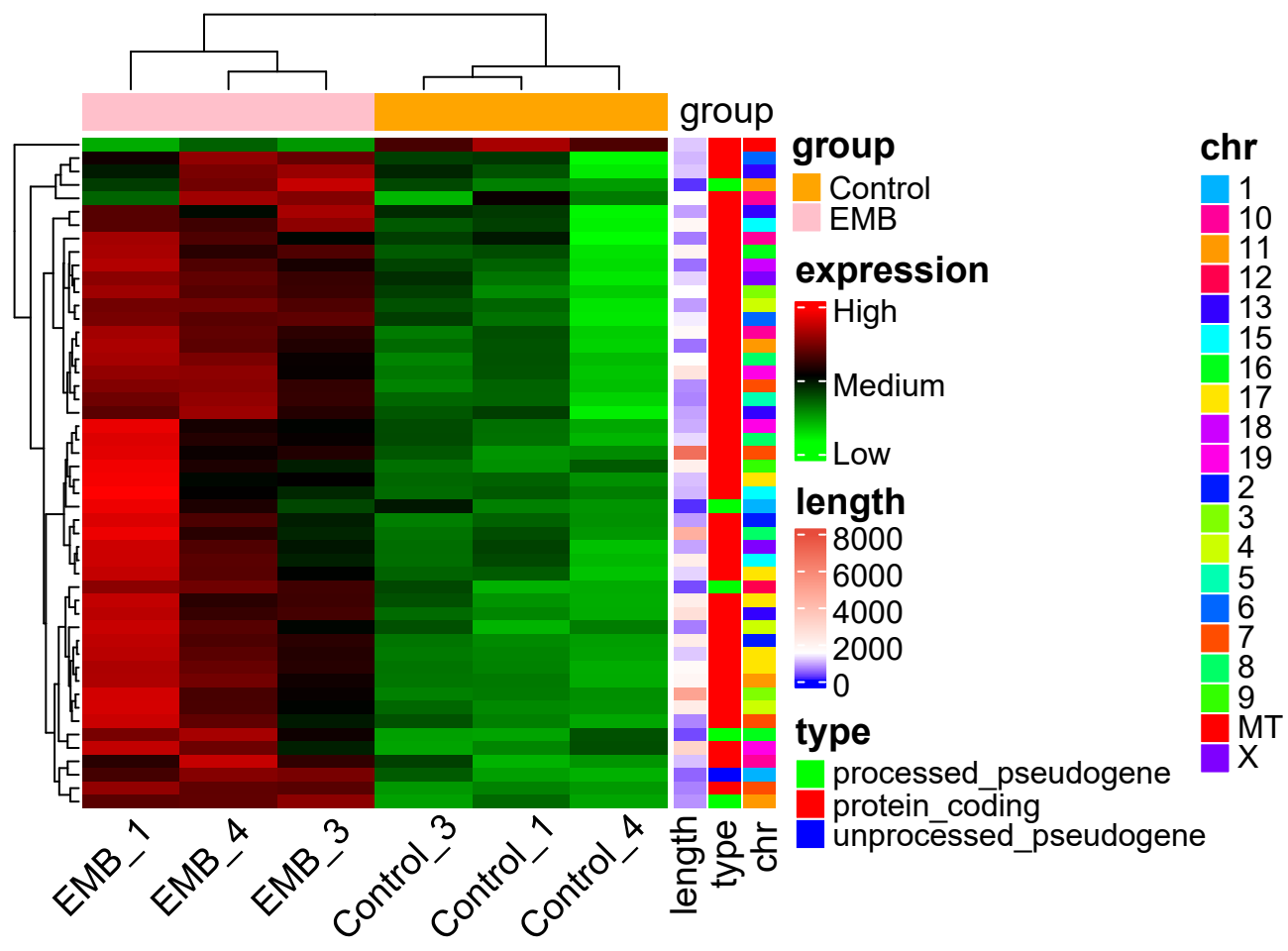

Supplement: Supplementary file 6 — Supplementary file6 Venn_heatmap (PDF 185 KB) [file 13577_2025_1342_MOESM6_ESM.pdf]
