# Supplementary material for: Ethambutol induces optic neuropathy through SDHB-mediated ferroptosis in retinal ganglion cells via Smad4 pathway
Source: Hum Cell. 2026 Jan 21;39(2):37. doi: 10.1007/s13577-025-01342-4 (PMC12823716; doi:10.1007/s13577-025-01342-4)
Supplement: Supplementary file 5 — Supplementary file5 Venn (PDF 123 KB) [file 13577_2025_1342_MOESM5_ESM.pdf]

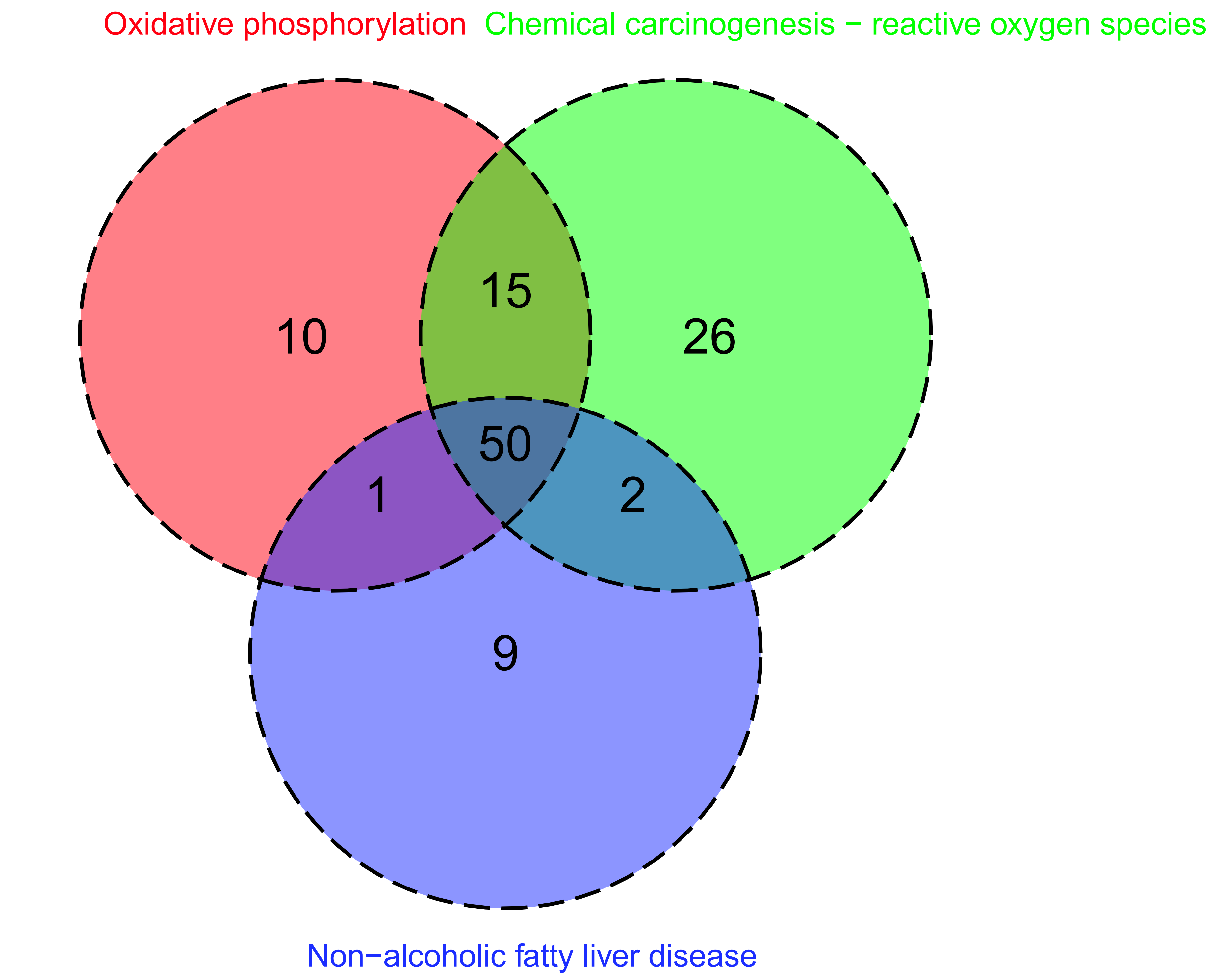
Oxidative phosphorylation
Chemical carcinogenesis − reactive oxygen species
10
15
26
50
1
2
9
Non−alcoholic fatty liver disease
