# Supplementary material for: Ethambutol induces optic neuropathy through SDHB-mediated ferroptosis in retinal ganglion cells via Smad4 pathway
Source: Hum Cell. 2026 Jan 21;39(2):37. doi: 10.1007/s13577-025-01342-4 (PMC12823716; doi:10.1007/s13577-025-01342-4)
Supplement: Supplementary file 7 — Supplementary file7 Western blot original data (PDF 354 KB) [file 13577_2025_1342_MOESM7_ESM.pdf]

Figure2 B

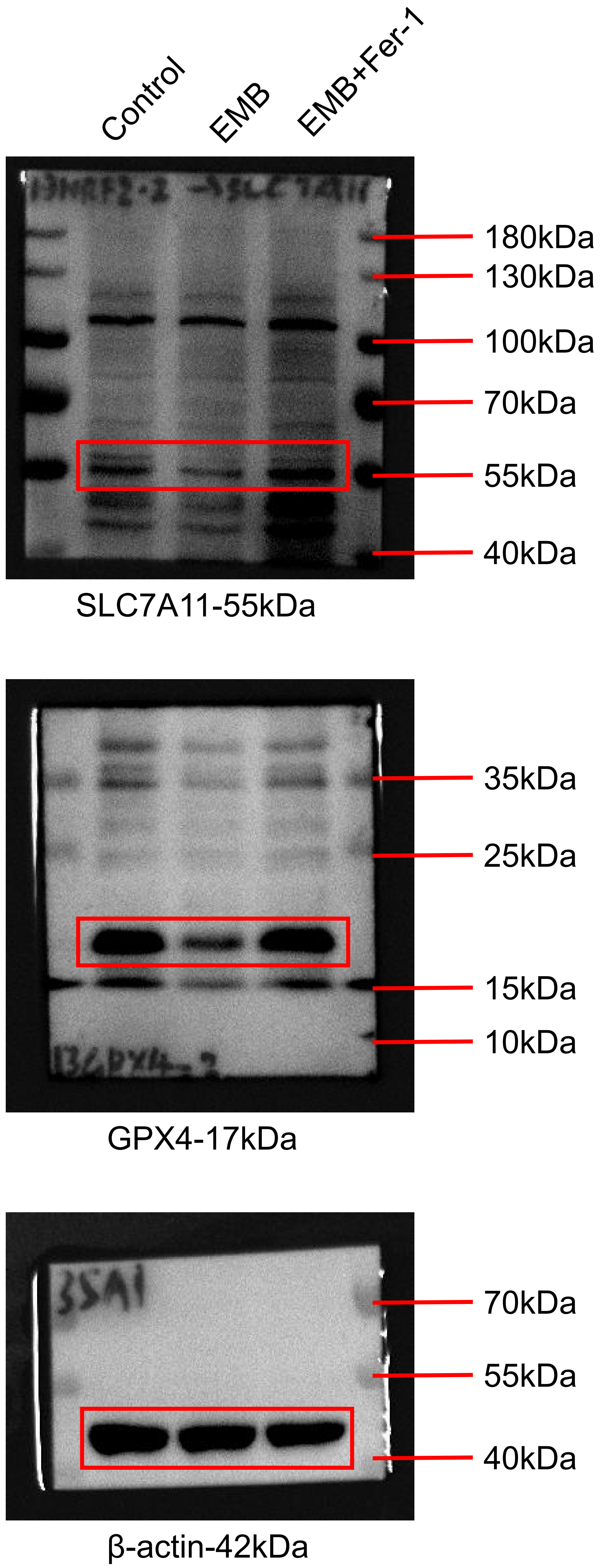

Figure2 C

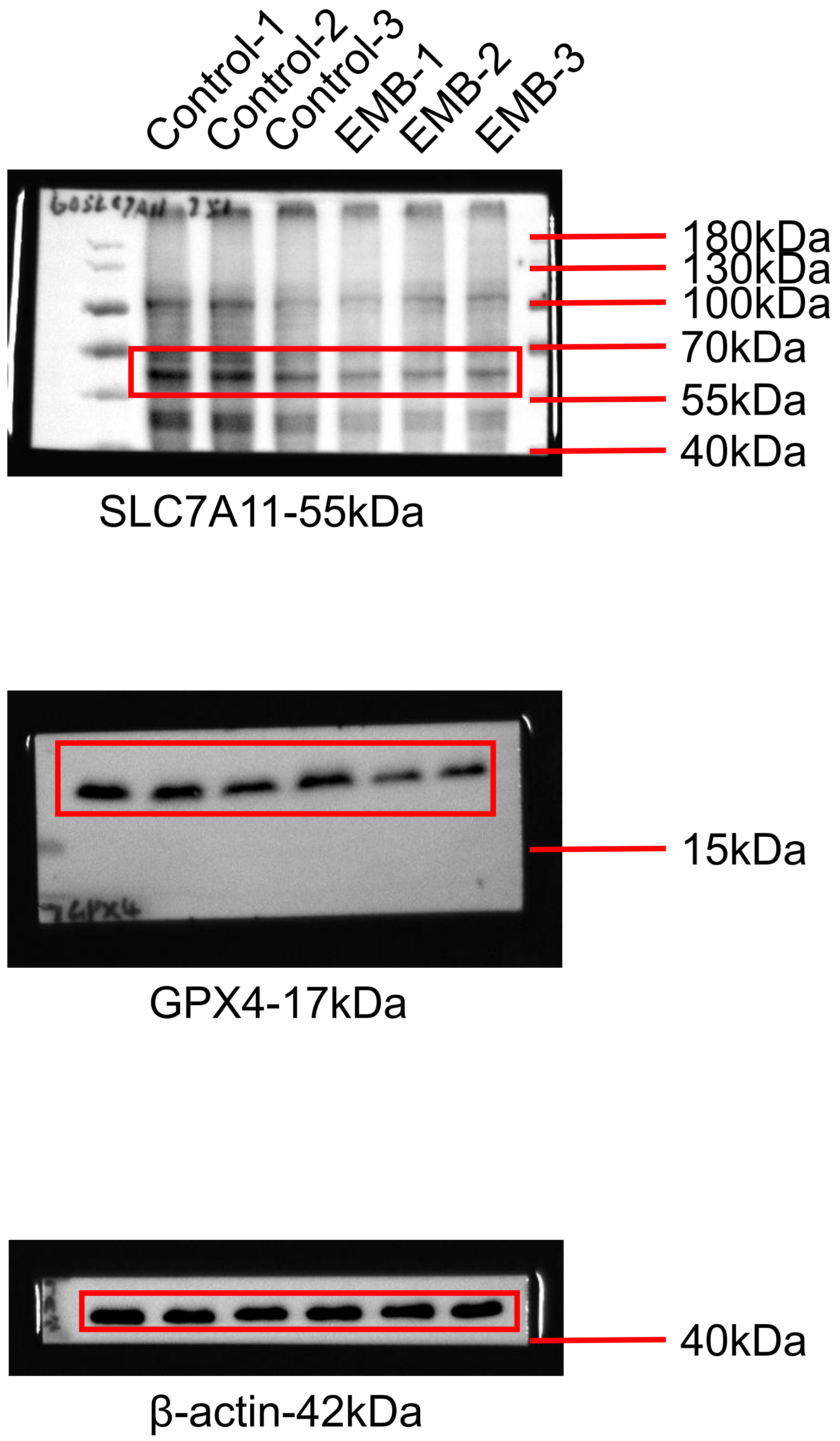

Figure4 C

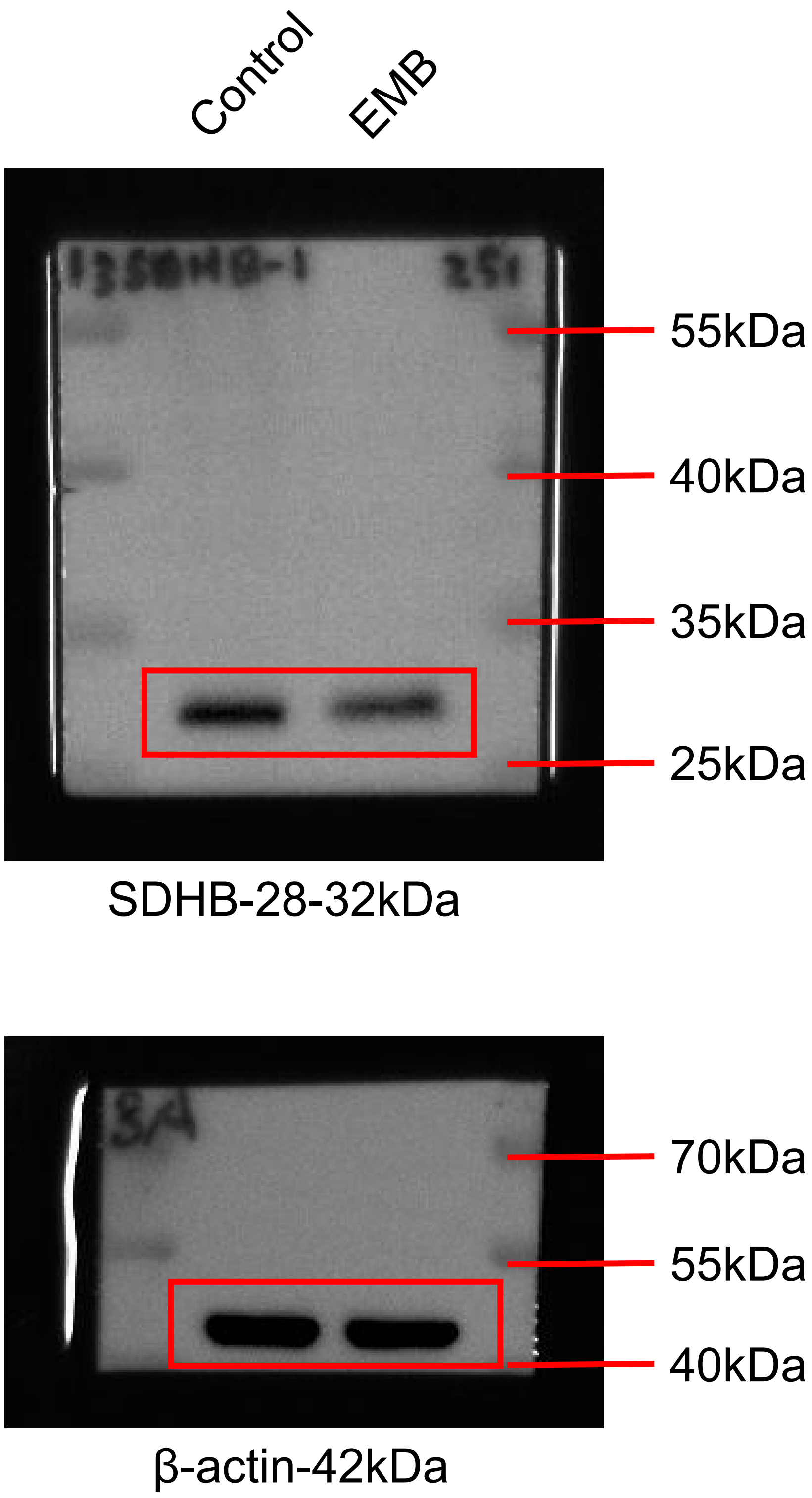

Figure4 D

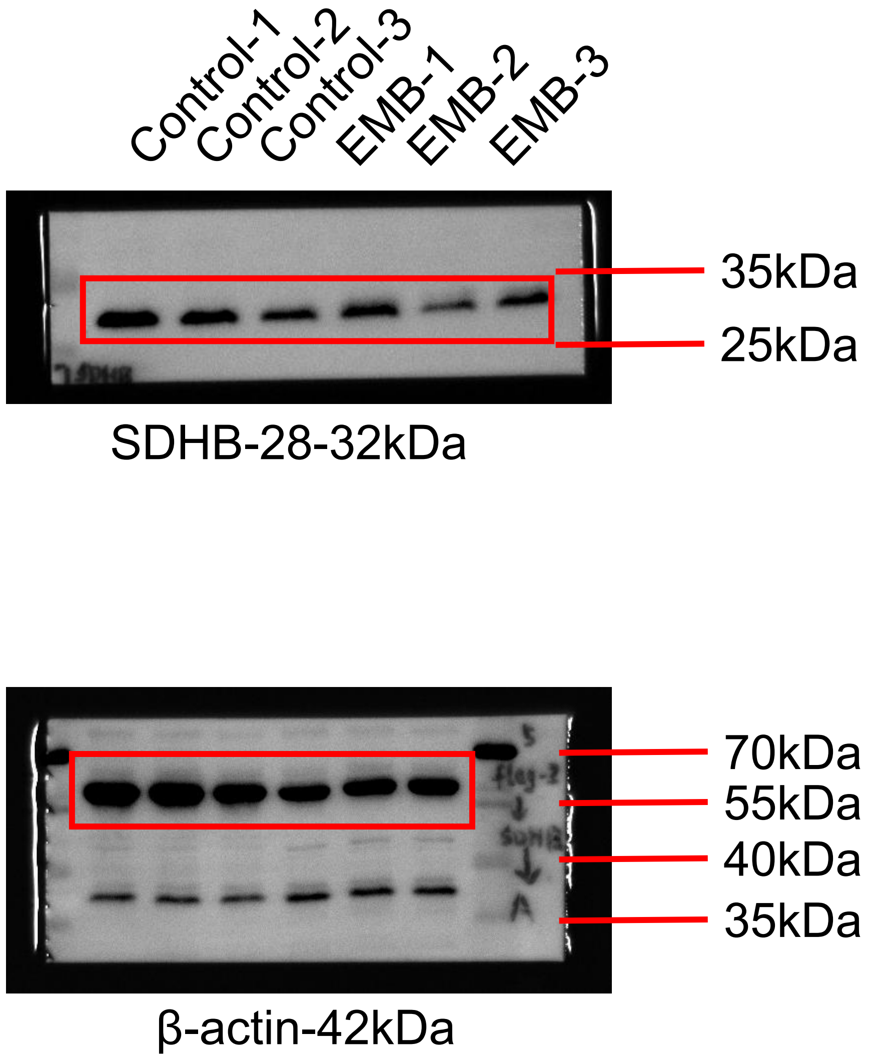

Figure5 A

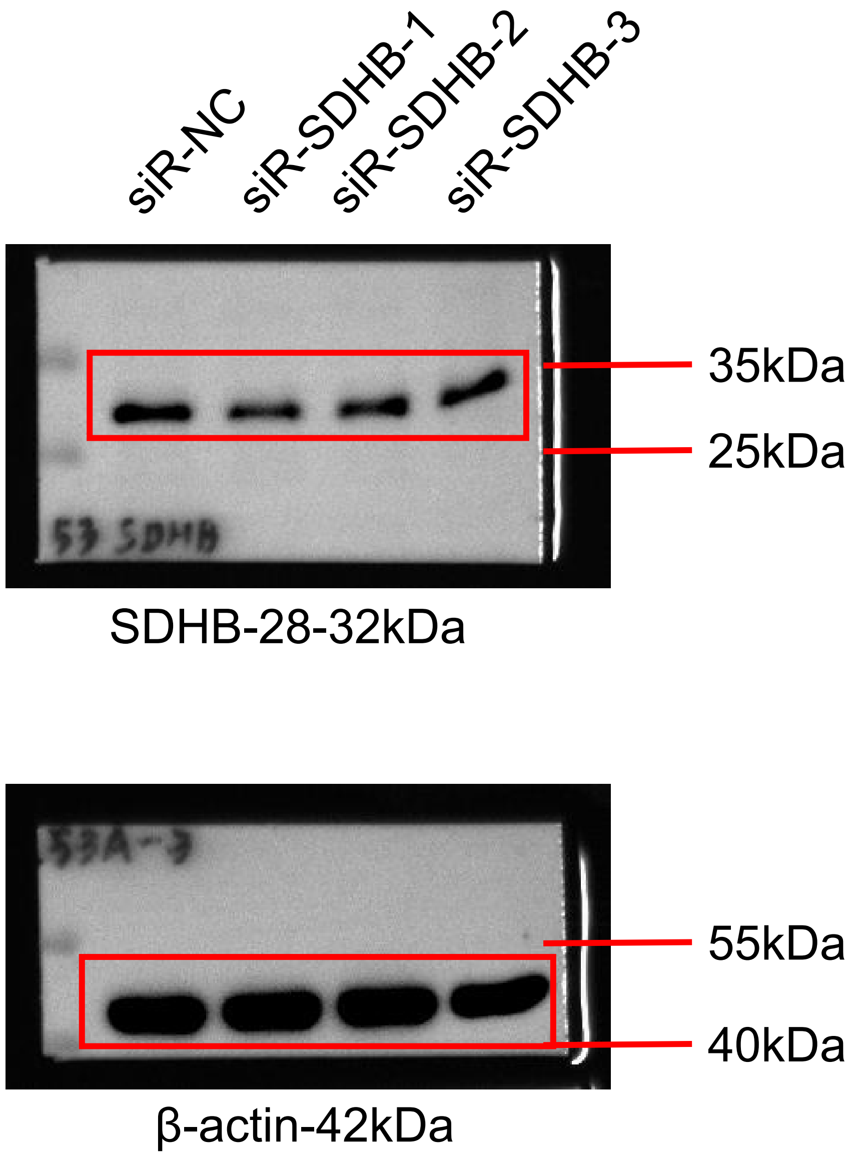

Figure5 B

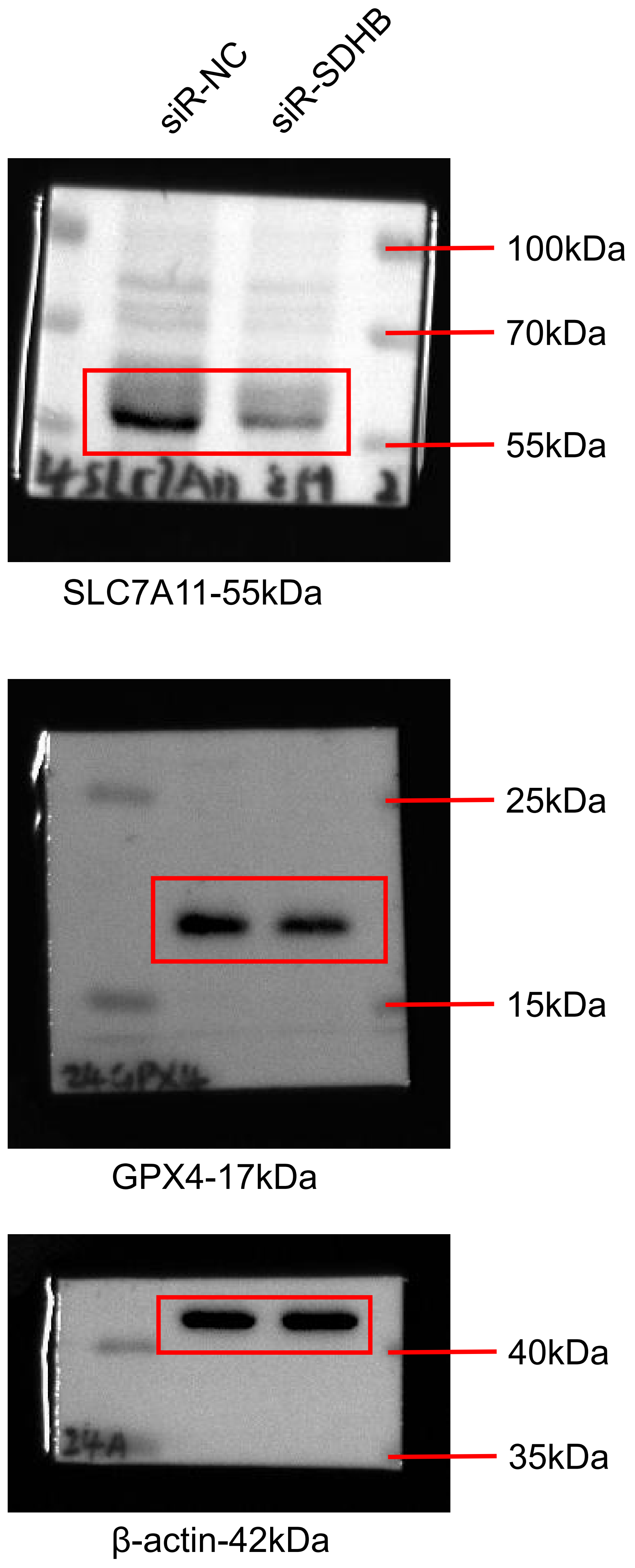

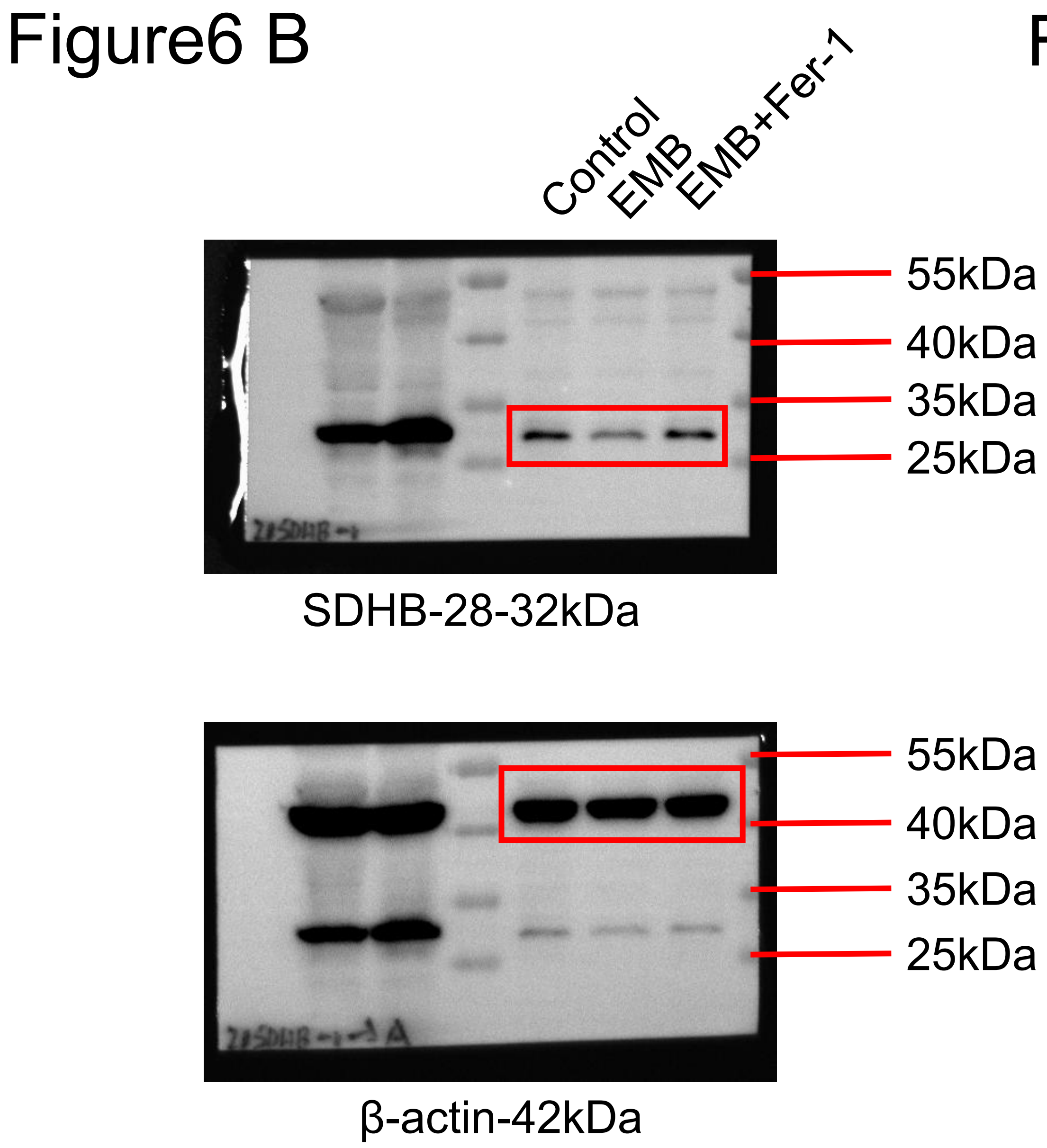

Figure6 B
Control
EMB
EMB+Fer-1
55kDa
40kDa
35kDa
25kDa
SDHB-28-32kDa
55kDa
40kDa
35kDa
25kDa
β-actin-42kDa

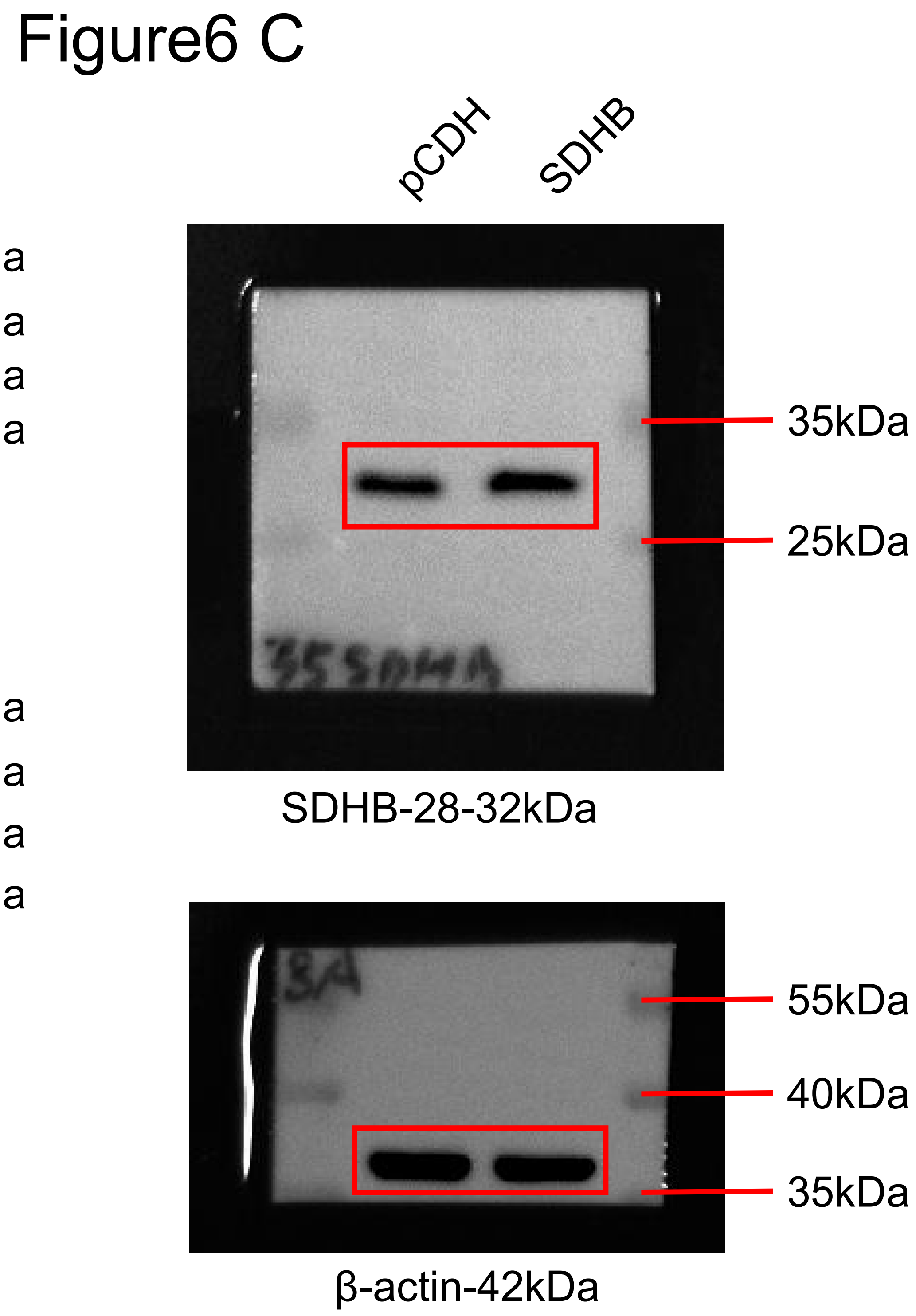

Figure6 C
pCDH
SDHB
35kDa
25kDa
SDHB-28-32kDa
55kDa
40kDa
35kDa
β-actin-42kDa

Figure6 D

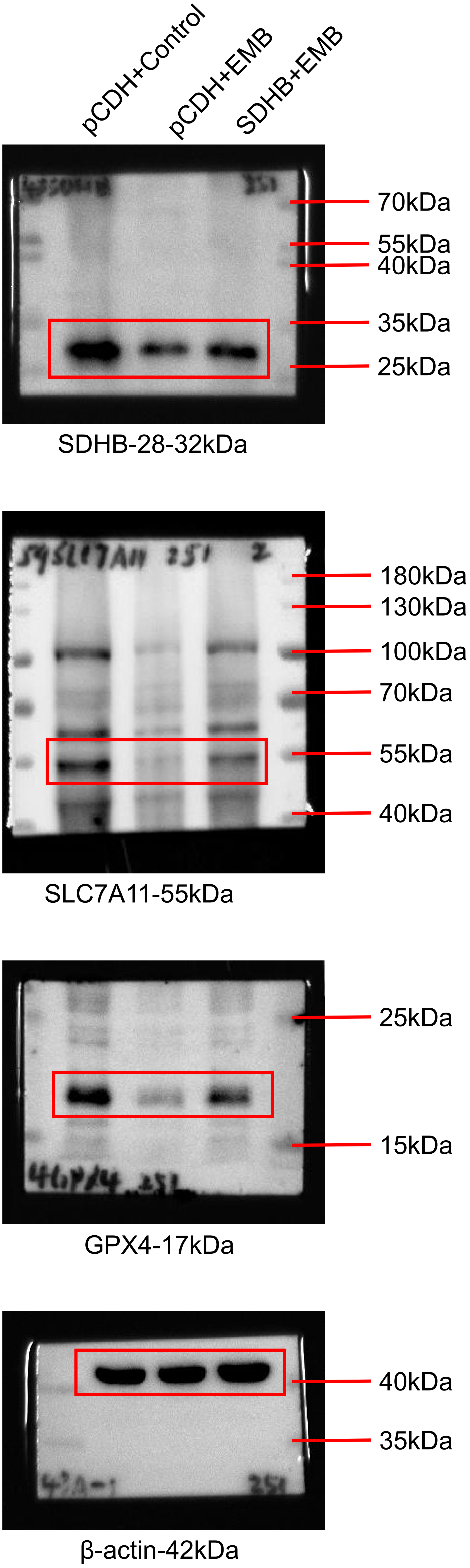
pCDH+Control
pCDH+EMB
SDHB+EMB
70kDa
55kDa
40kDa
35kDa
25kDa
SDHB-28-32kDa
180kDa
130kDa
100kDa
70kDa
55kDa
40kDa
SLC7A11-55kDa
25kDa
15kDa
GPX4-17kDa
40kDa
35kDa
β-actin-42kDa

Figure7 A

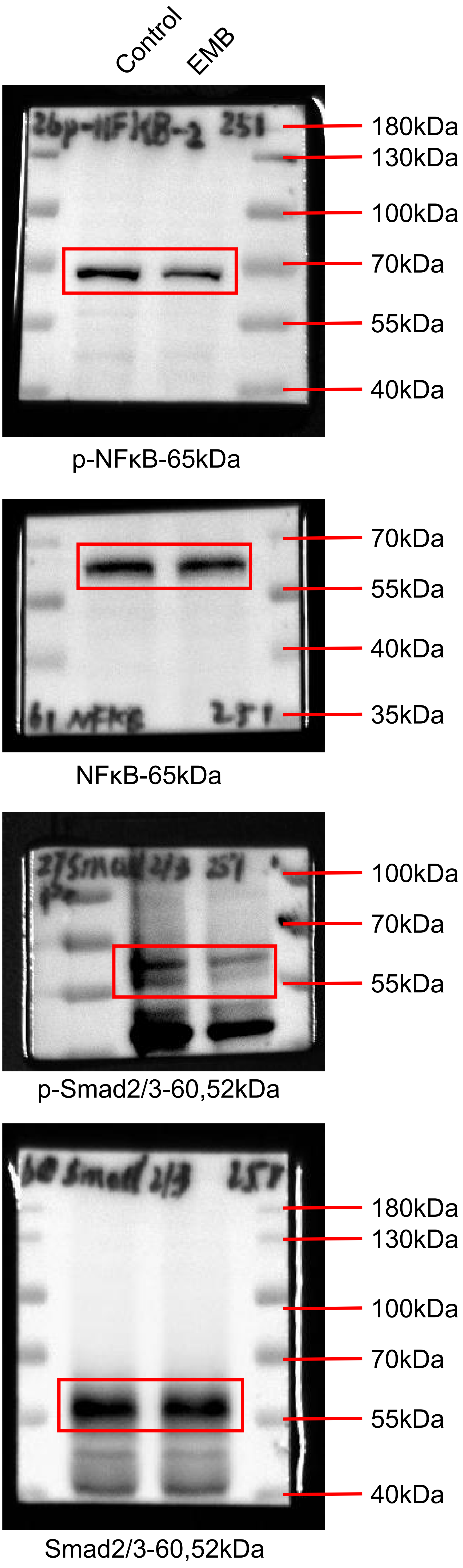
Control
EMB
180kDa
130kDa
100kDa
70kDa
55kDa
40kDa
p-NFκB-65kDa
70kDa
55kDa
40kDa
35kDa
NFκB-65kDa
100kDa
70kDa
55kDa
p-Smad2/3-60,52kDa
180kDa
130kDa
100kDa
70kDa
55kDa
40kDa
Smad2/3-60,52kDa

Figure7 A

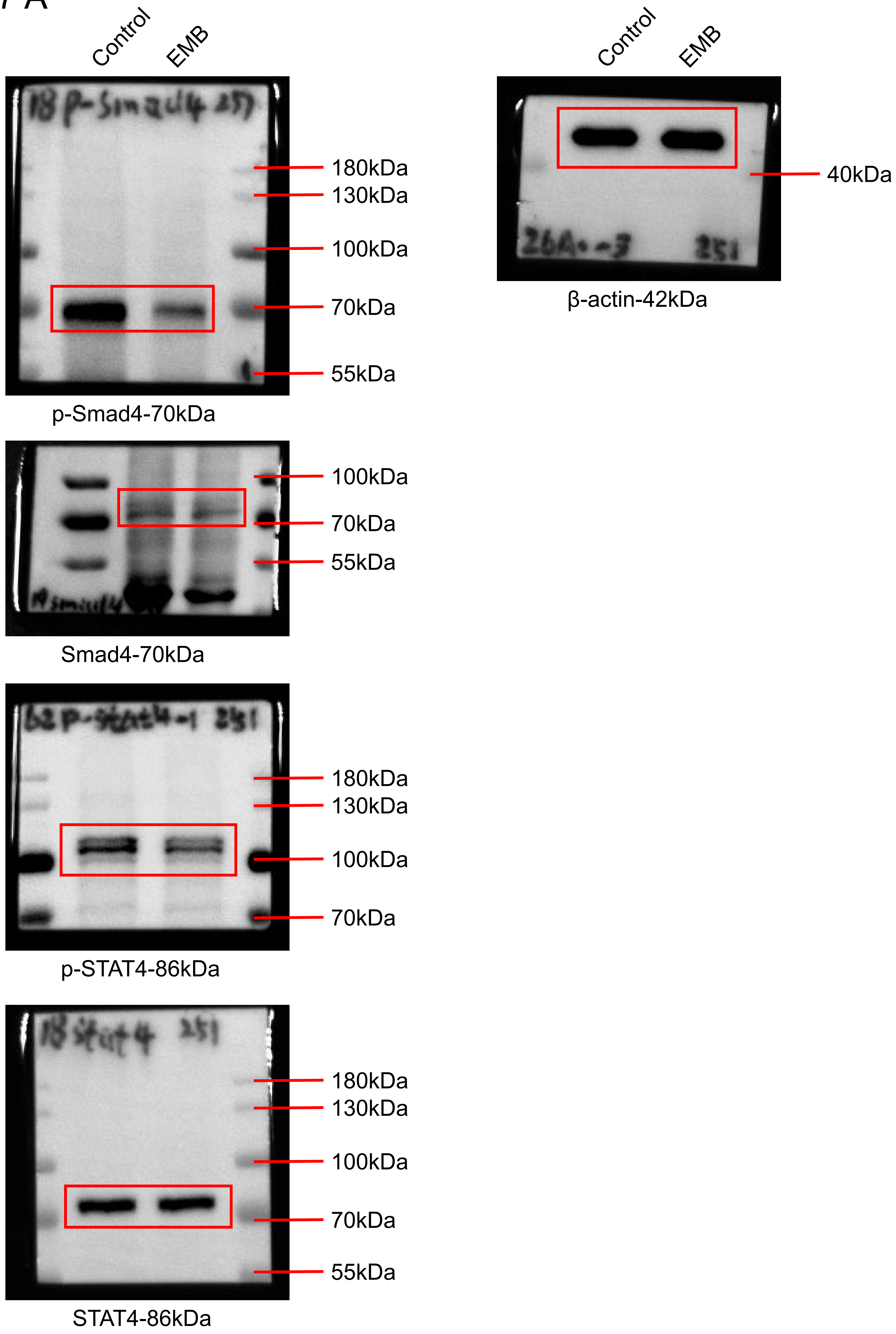
Control
EMB
180kDa
130kDa
100kDa
70kDa
55kDa
p-Smad4-70kDa
Smad4-70kDa
p-STAT4-86kDa
STAT4-86kDa
40kDa
β-actin-42kDa

Figure7 C

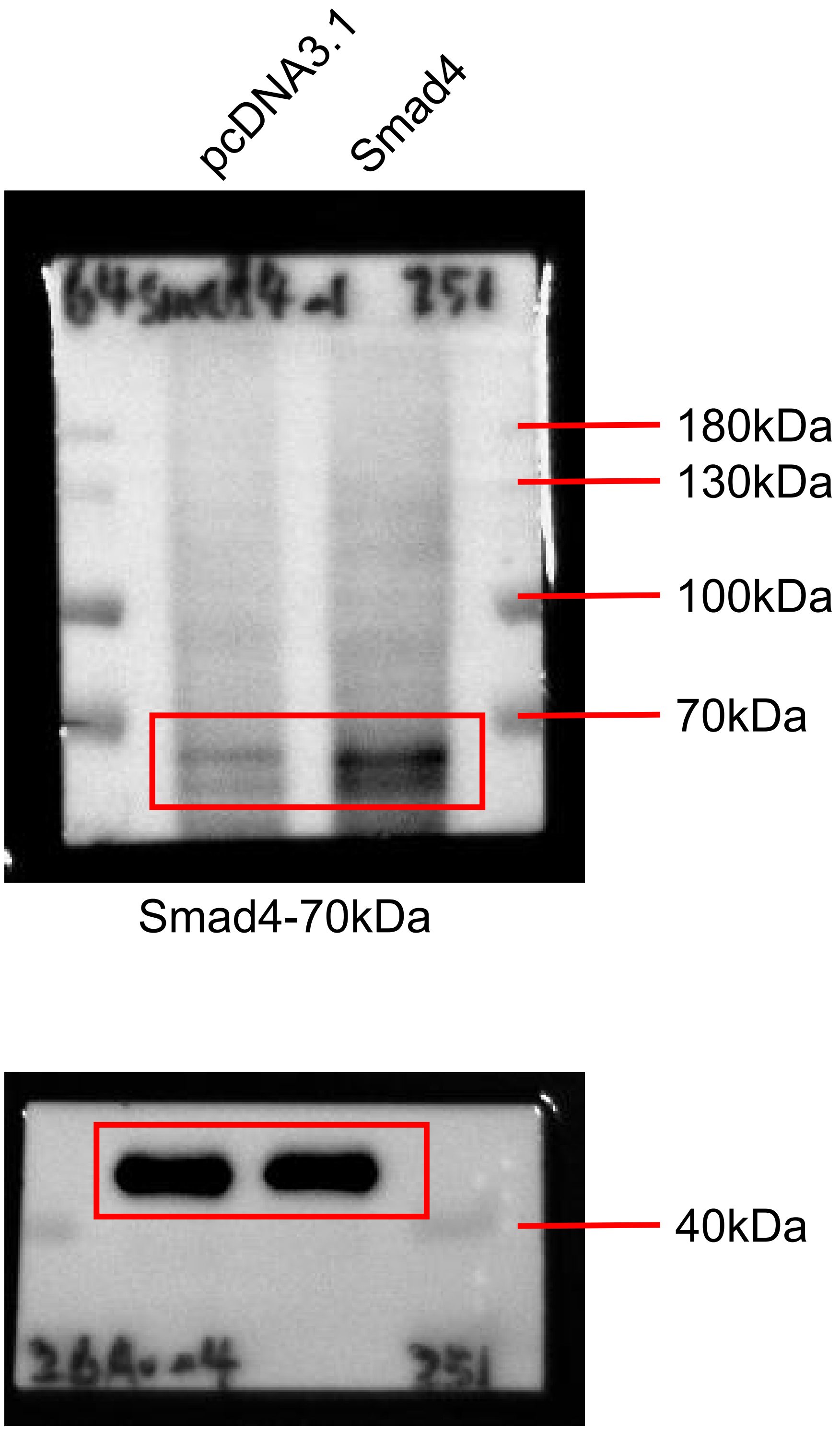

Smad4-70kDa

β-actin-42kDa

Figure7 E

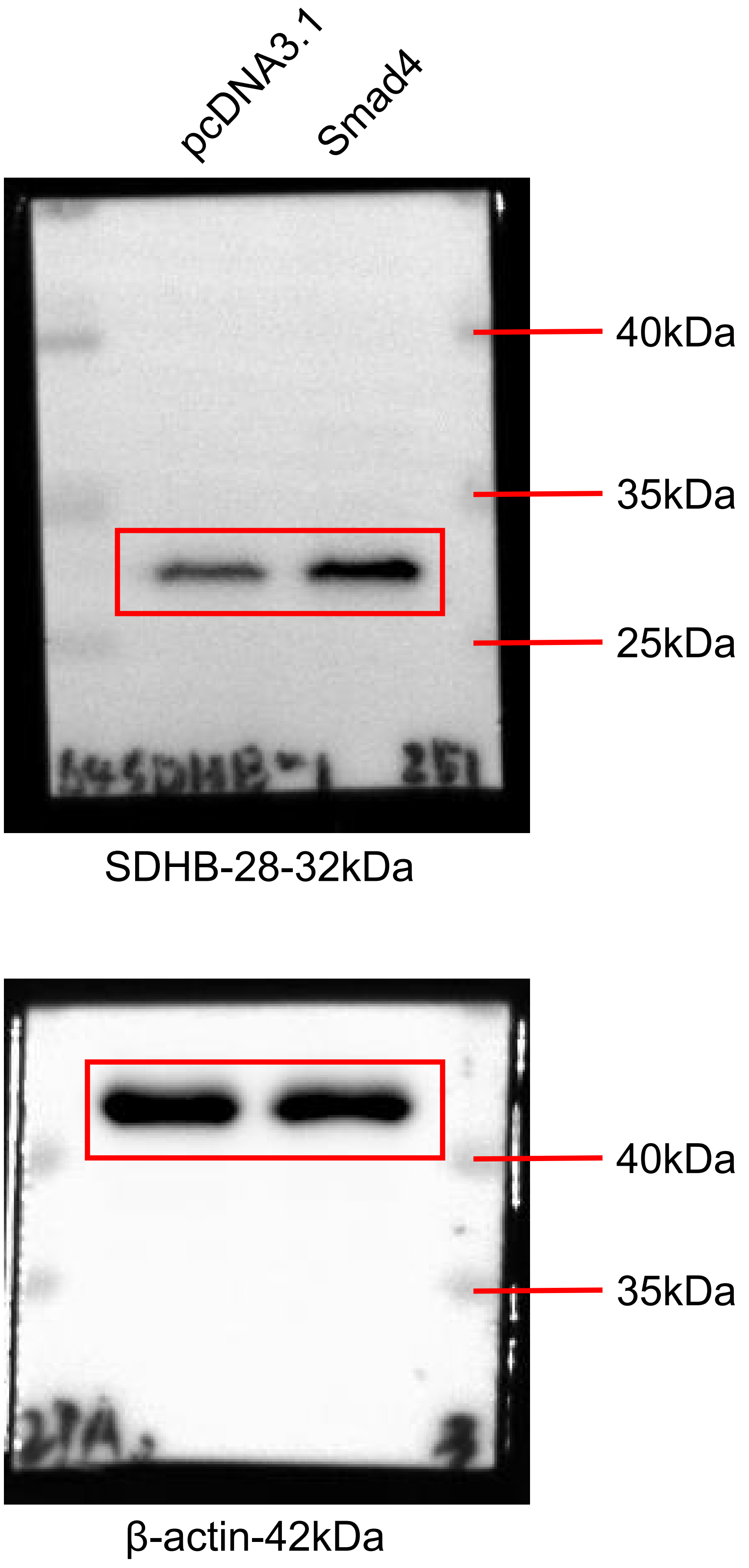
pcDNA3.1
Smad4
40kDa
35kDa
25kDa
SDHB-28-32kDa
40kDa
35kDa
β-actin-42kDa
